# *Akkermansia muciniphila* counteracts the deleterious effects of dietary emulsifiers on microbiota and host metabolism

**DOI:** 10.1136/gutjnl-2021-326835

**Published:** 2023-01-16

**Authors:** Noëmie Daniel, Andrew T Gewirtz, Benoit Chassaing

**Affiliations:** 1 Team “Mucosal Microbiota in Chronic Inflammatory Diseases”, Institut Cochin, INSERM U1016, CNRS UMR 8104, Université Paris Cité, Paris, France; 2 Institute for Biomedical Sciences, Center for inflammation, Immunity and Infection, Digestive Disease Research Group, Georgia State University, Atlanta, Georgia, USA

**Keywords:** MUCUS, INFLAMMATION, INTESTINAL MICROBIOLOGY, PROBIOTICS

## Abstract

**Background:**

Accumulating evidence indicates that some non-absorbed food additives, including emulsifiers carboxymethylcellulose (CMC) and polysorbate 80 (P80), can negatively impact intestinal microbiota, leading to microbiota encroachment, chronic low-grade intestinal inflammation and, subsequently, promotion of metabolic dysregulations. Detrimental impacts of emulsifier consumption on gut microbiota include depletion of the health-associated mucus-fortifying bacteria, *Akkermansia muciniphila*.

**Objective:**

Investigate, in mice, the potential of administration of exogenous *A. muciniphila* as a means to protect against detrimental impacts of emulsifiers.

**Results:**

Daily oral administration of *A. muciniphila* prevented phenotypic consequences of consumption of both CMC and P80, including hyperphagia, weight gain and dysglycaemia. *A. muciniphila* administration also counteracted the low-grade intestinal inflammation-induced CMC and P80. Furthermore, *A. muciniphila* supplementation prevented the proximal impacts of CMC and P80 on gut microbiota that are thought to drive low-grade chronic inflammation and metabolic dysregulations. Specifically, *A. muciniphila* prevented alterations in species composition and encroachment of gut microbiota that were otherwise induced by CMC and P80. Remarkably, we finally report that CMC and P80 altered the colonic transcriptome, while *A. muciniphila* largely protected against these alterations.

**Conclusion:**

Daily administration of *A. muciniphila* protects against the detrimental impact of emulsifiers on both the microbiota and host. These results support the notion that use of *A. muciniphila* as a probiotic can help maintain intestinal and metabolic health amidst the broad array of modern stresses that can promote chronic inflammatory diseases.

**WHAT IS ALREADY KNOWN ON THIS TOPIC**

- Previous findings reported that commonly used dietary emulsifiers alter the intestinal microbiota and promote chronic intestinal inflammation and metabolic dysregulations.
- Microbiota encroachment is a central step in emulsifier-induced detrimental consequences.
- *Akkermansia muciniphila* is a next-generation beneficial probiotic able to reinforce the intestinal barrier and prevent metabolic dysregulations.

**WHAT THIS STUDY ADDS**

- *A. muciniphila* supplementation prevents metabolic dysregulations that are otherwise induced by emulsifier consumption.
- *A. muciniphila* prevent alterations in species composition and encroachment of gut microbiota that are otherwise induced by carboxymethylcellulose and polysorbate 80.
- Dietary emulsifiers alter the colonic transcriptome, while *A. muciniphila* largely protects against these alterations.

**HOW THIS STUDY MIGHT AFFECT RESEARCH, PRACTICE OR POLICY**

- Our findings underlie *A. muciniphila* as a therapeutic approach to protect against the detrimental impact of emulsifiers on both the microbiota and host.
- *A. muciniphila* might help to maintain intestinal and metabolic health in the context of modern stresses that normally promote chronic inflammatory diseases.

## Introduction

Humanity is faced with a stark increase in the constellation of metabolic disorders referred to as metabolic syndrome, the cardinal features of which include obesity and insulin resistance. Metabolic syndrome is associated with alterations in gut microbiota composition.^1^ Faecal microbiota transplant studies in mice and humans argue that such alterations do not merely mark, but rather promote, dysregulated metabolism.^2^ While mechanisms by which altered microbiota promote metabolic dysregulation are not entirely clear, the inverse correlation of microbiota-epithelial distance with extent of dysglycaemia^3^ suggests an important role for microbiota that encroach into the normally near-sterile inner mucus layer, perhaps reflecting that such encroaching bacteria promote low-grade inflammation, which can subsequently dysregulate metabolism.

A variety of factors can induce microbiota dysbiosis and encroachment, including consumption of a high-fat low-fibre ‘western-style’ diet^4^ and the class of food additives known as emulsifiers.^5^ Emulsifiers are incorporated into many processed foods to extend shelf life and improve organoleptic properties^6 7^ and they are suspected to be a significant driver of the association of consumption of ultraprocessed foods consumption with development of chronic inflammatory diseases.^8 9^ Some emulsifiers, for example, lecithin, are natural dietary components, while others, including carboxymethylcellulose (CMC) and polysorbate 80 (P80) are human synthetic creations. Consumption of CMC and P80 alter gut microbiota composition and induce microbiota encroachment in mice, and a recent report suggests CMC acts in a similar manner in humans.^10^ Such microbiota dysbiosis and encroachment associate with chronic low-grade intestinal inflammation that manifest as metabolic dysregulations in wild-type mice and potentiation of colitis in mice genetically prone to this disorder.^5^ Studies using in vitro microbiota models suggest that CMC and P80 may perturb host–microbiota homeostasis as a result of their direct action on microbiota.^11 12^

The ubiquity of emulsifiers, and other additives, in processed foods, which provide a significant portion of human food consumption, makes avoiding these additives challenging. Hence, as a possible countermeasure to emulsifiers, we turned to the bacterium *Akkermansia muciniphila*, which exhibits reduced abundance in metabolic syndrome and, moreover, can protect against this state when exogenously administered.^13 14^ *A. muciniphila* fortifies the mucosal barrier by stimulating mucus production, leading to a thicker mucus layer under a mucus-disruptive high-fat diet^15 16^ and, furthermore, by inducing production of antimicrobial peptides such as Reg3γ.^17^ Beneficial effects of *A. muciniphila* can be observed when using the intact bacteria, its pasteurised (but not autoclaved) form, or its outer membrane and secreted proteins,^15 18–20^ suggesting that its beneficial properties are linked to surface/secreted molecules, rather than its metabolic activity, including mucus digestion. A pilot clinical study of *A. muciniphila* suggested benefits in humans, including a trend towards lowered fat-mass gain and decreased hip circumference, enhanced insulin sensitivity and reductions in endotoxaemia and inflammation in overweight subjects.^21^ Thus, the goal of this study was to investigate the potential of *A. muciniphil*a to prevent emulsifier disturbance of host-microbiota homeostasis as well as its impact on low-grade inflammation and metabolism. We found that daily administration of *A. muciniphila* protects mice from emulsifier-induced metabolic dysregulations and the low-grade intestinal inflammation thought to drive this state. Furthermore, *A. muciniphila* prevents emulsifier-induced shifts in microbiota composition and localisation, as well as protects against colonic transcriptome alterations. Such ability of *A. muciniphila* supports its use as a countermeasure to combat modern stressors that perturb host–microbiota interactions to promote metabolic syndrome and other chronic inflammatory diseases.

## Results

### Impact of dietary emulsifier consumption on the faecal abundance of *A. muciniphila*

**Figure 1:**
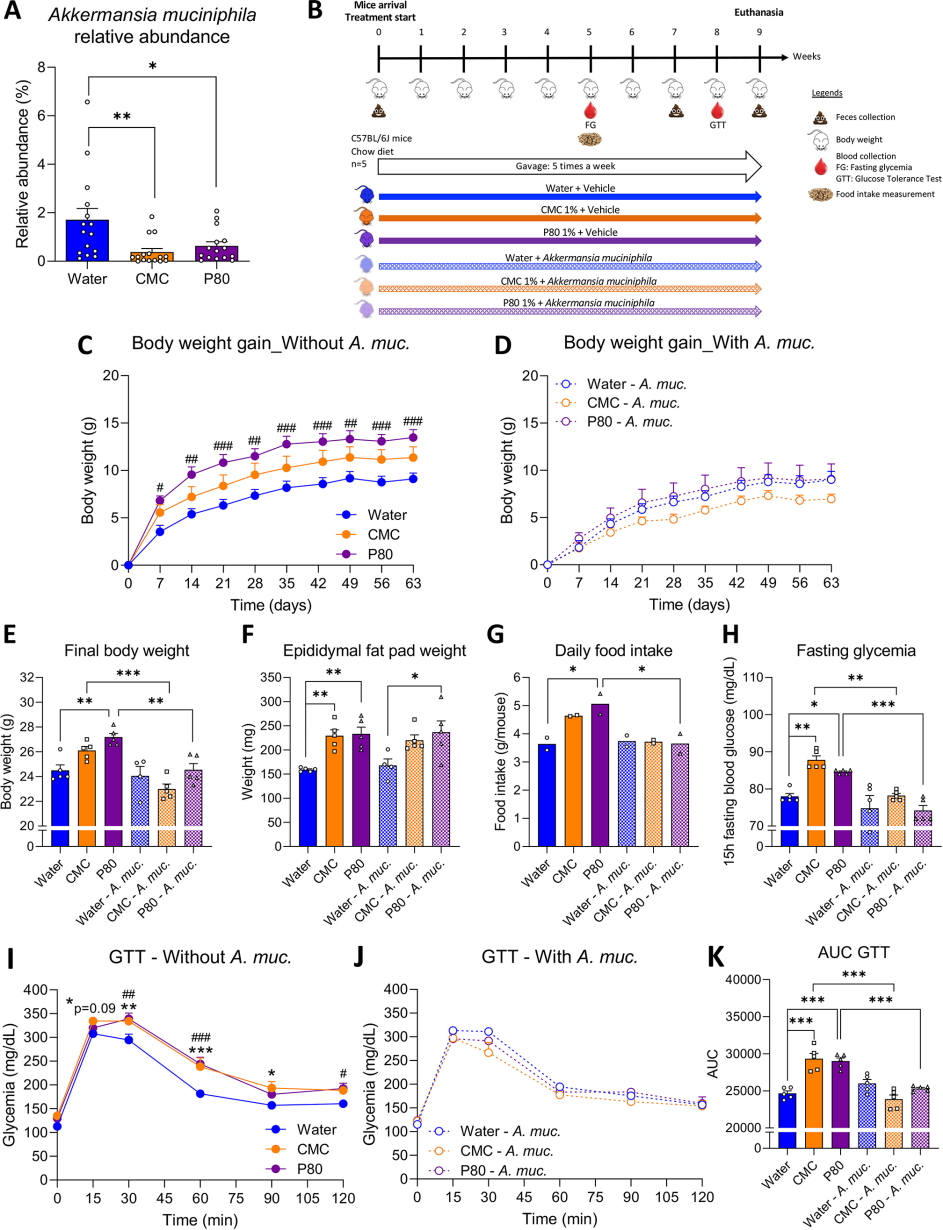
*Akkermansia muciniphila* administration prevented emulsifier-induced metabolic deregulations. (A) Relative abundance of faecal *A. muciniphila i*n mice chronically exposed to drinking water, CMC 1.0% or P80 1.0%. (B) Schematic representation of the experimental design. Mice were exposed to drinking water (blue) containing 1.0% of CMC (orange) or p80 (purple) for 9 weeks, and gavaged 5 days per week with either vehicle (sterile PBS, solid lines and bars) or *A. muciniphila* (*A. muc.*, hatched lines and bars). Body weight gain over time of mice orally receiving (C) vehicle or (D) *A. muciniphila*. (E) Final body weight, (F) epididymal fat pad weight, (G) daily food intake measurement and (H) 15 hours fasting blood glucose level. (I–K) At week 8, mice were 5 hours fasted, challenged with an intraperitoneal bolus of glucose (2 g/kg of body weight). Glycaemic response was measured after 15, 30, 60, 90, 120 min in mice receiving (I) vehicle or (J) *A. muciniphila*. (K) Areas under the curves obtained from the glucose tolerance test. Data are represented as means±SEM. n=4–5. For bar graphs, statistical analyses were performed using a one-way ANOVA followed by a Bonferroni post hoc test and significant differences were recorded as follows: *p<0.05, **p<0.01, ***p<0.001. For line charts, a two-way ANOVA or a mixed model (if missing values) was performed, followed by a Bonferroni post hoc test, and significant differences were recorded as follows: CMC vs water, *p<0.05, **p<0.01, ***p<0.001; P80 vs water, ^#^p<0.05, ^##^p<0.01, ^###^p<0.001. Exact p values for trends (0.05≤p<0.10) are recorded on graphs for additional indication. ANOVA, analysis of variance; CMC, carboxymethylcellulose; P80, polysorbate 80.

Some dietary emulsifiers, including the synthetic compounds CMC and P80, have the potential to disrupt host–microbiota interactions resulting in low-grade intestinal inflammation and dysregulated metabolism.^5 11 15^ Impacts of these emulsifiers on microbiota include numerous alterations in relative species abundance, as well as depletion of beneficial bacteria.^5 10^ In accord with this notion, our analysis here of microbiota composition of emulsifier-treated mice revealed that chronic consumption of either CMC or P80 significantly decreased relative faecal abundance of *A. muciniphila* (figure 1A). Considering the well-established probiotic health potential of this bacterium,^16–20^ including its ability to fortify mucus, we hypothesised that supplementing microbiota *via* exogenous administration of *A. muciniphila* might counteract deleterious impacts of emulsifier consumption.

### *Akkermansia muciniphila* administration prevents emulsifier-induced metabolic dysregulations

C57/Bl6 mice were exposed to water, CMC (1%) or P80 (1%) for 9 weeks, while concomitantly treated with either phosphate buffered saline (PBS) - vehicle or *A. muciniphila* by oral gavage 5 days per week (figure 1B), using the #BAA-835 (ATCC) *A. muciniphila* strain, isolated by Derrien *et al*.^13^ Culture purity was confirmed, after bacteria growth, washing and aliquoting (*cf.* Method section for details), via 16S rRNA gene amplification and sequencing (online supplemental figure S1A). We subsequently used these verified *A. muciniphila* aliquots to treat mice daily with 2.5×10^8^ CFU, an approach that mildly, but nonetheless significantly, increased *A. muciniphila* faecal relative abundance, as assessed by qPCR approach (online supplemental figure S1B).

10.1136/gutjnl-2021-326835.supp4 Supplementary data

As previously reported, P80 induced a greater body weight gain in mice compared with the control group, with a similar trend observed for CMC-treated mice (figure 1C). This body weight gain was abrogated in mice receiving *A. muciniphila*, with all *A. muciniphila*-treated group presenting similar final body weights as non-emulsifier-treated control mice (figure 1D, E). In contrast, *A. muciniphila* did not alter body weight in water-treated animals (figure 1D, E, p=0.80), consistent with the possibility that *A. muciniphila* was ameliorating a dysbiotic state rather than directly impacting host metabolism. Impact of emulsifiers on body weight were generally associated with impact on fat pad weight, as presented figure 1F. Furthermore, *A. muciniphila* administration completely abrogated CMC and P80’s induction of overeating and hyperglycaemia (figure 1G, H). To better evaluate the impact of *A. muciniphila* administration on glucose homoeostasis, an intraperitoneal glucose tolerance test (GTT) was performed after 8 weeks of emulsifier exposure. As presented in figure 1I, J both CMC- and P80-treated mice exhibited significant alteration in their glucose excursion curve (figure 1I), while no differences were observed in mice receiving *A. muciniphila* (figure 1J), with both CMC-treated and P80-treated groups aligning with the water-only treated group. Measure of areas under the curves further supported that emulsifier-induced glucose intolerance was fully prevented with daily administration of *A. muciniphila* (figure 1K). Altogether, these data demonstrate that *A. muciniphila* administration was sufficient to largely prevent emulsifier promotion of metabolic dysregulations.

### *A. muciniphila* administration prevents emulsifier-induced low-grade intestinal inflammation

**Figure 2:**
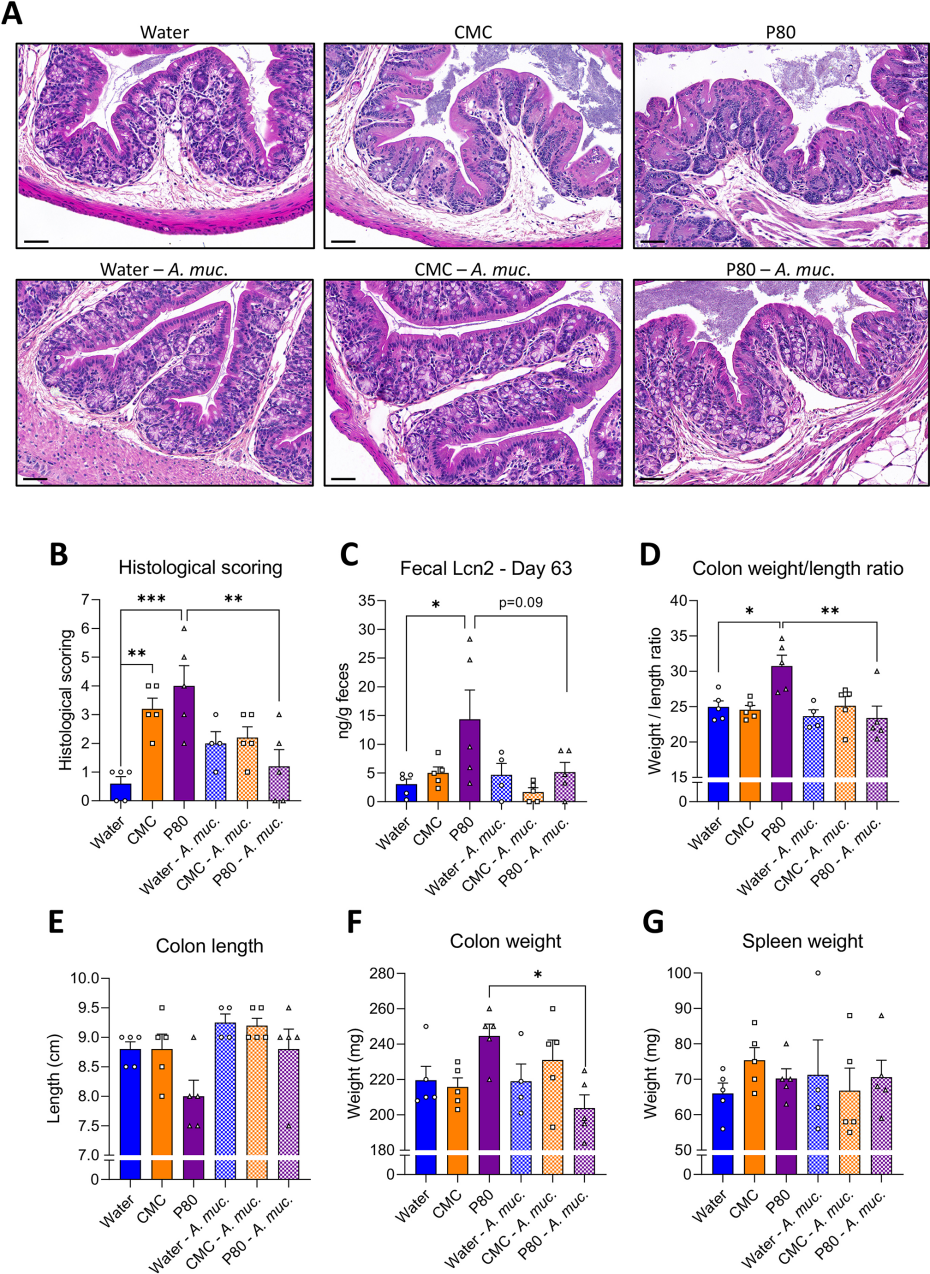
*Akkermansia muciniphila* administration prevents emulsifier-induced low-grade intestinal inflammation. Mice were exposed to drinking water (blue) containing 1.0% of CMC (orange) or P80 (purple) for 9 weeks, and gavaged 5 days per week with either vehicle (sterile PBS, solid bars) or *A. muciniphila* (*A. muc.*, hatched bars). (A) Representative images of (B) the histopathological score of H&E-stained colonic sections; scale bar, 100 µm. (C) Faecal lipocalin-2 (Lcn2) level at day 63, (D) weight/length ratio, (E) colon length, (F) colon weight, and (G) spleen weight. Data are represented as means±SEM. n=4–5. Statistical analyses were performed using a one-way ANOVA followed by a Bonferroni post hoc test and significant differences were recorded as follows: *p<0.05, **p<0.01, ***p<0.001. Exact p values for trends (0.05≤p< 0.10) are recorded on graphs for additional indication. ANOVA, analysis of variance; CMC, carboxymethylcellulose; P80, polysorbate 80.

The negative impacts of emulsifiers on metabolism are thought to be driven by low-grade intestinal inflammation, which can be assayed by histopathological analysis, measure of inflammatory markers such as lipocalin-2 (Lcn2) and may manifest in gross morphological changes in colon and/or spleen. Accordingly, consumption of both CMC and P80 resulted in subtle but nonetheless histopathologically evident colon inflammation, particularly increased numbers of inflammatory cells infiltrating the mucosa and the submucosa (figure 2A, B, online supplemental table S1). Other indices of inflammation were more variable in that P80 also induced elevations in faecal Lcn2 and colon weight/length ratio, while CMC induced mild splenomegaly (figure 2C–G). *A. muciniphila* by itself did not significantly impact these parameters in water-treated mice. However, induction of low-grade inflammation by both CMC and P80 was abrogated by administration of *A. muciniphila*, suggesting this bacterium may broadly prevent negative impacts of emulsifiers.

10.1136/gutjnl-2021-326835.supp1 Supplementary data

### *A. muciniphila* administration prevents emulsifier-induced alterations in microbiota composition

**Figure 3:**
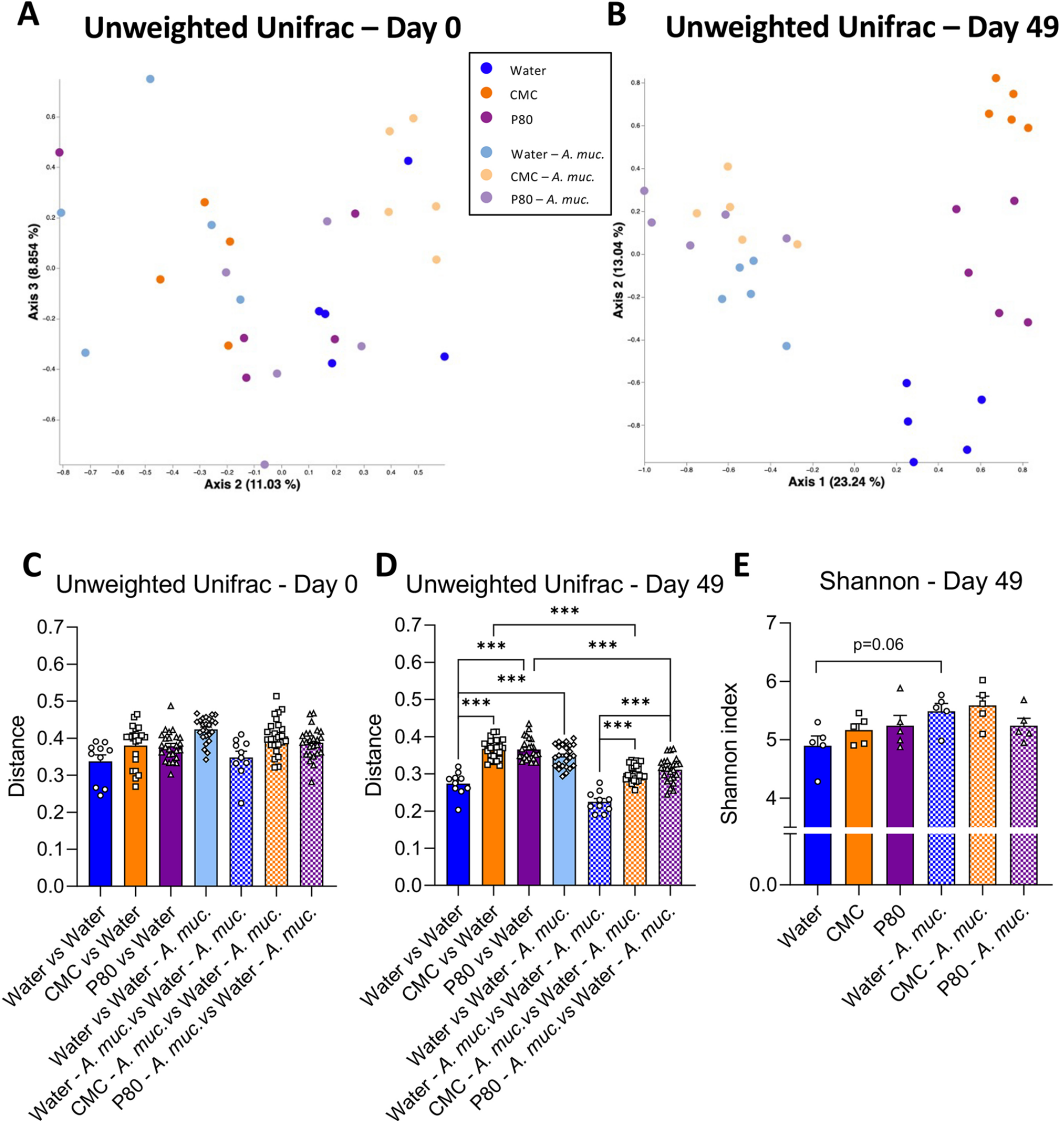
*Akkermansia muciniphila* administration dampens emulsifier-induced alterations in microbiota composition. Mice were exposed to drinking water (blue) containing 1.0% of CMC (orange) or P80 (purple) for 9 weeks, and gavaged 5 days per week with either sterile vehicle (sterile PBS, solid bars) or *A. muciniphila* (*A. muc.*, hatched bars). Bacterial DNA was extracted from faeces collected at days 0 and 49 and subjected to 16S rRNA gene sequencing. (A, B) Principal coordinates analysis (PcoA) of the unweighted Unifrac matrix of microbiota assessed by 16S rRNA gene sequencing at days (A) 0 and (B) 49. Each dot represents an individual animal and is colour coded (blue, water; orange, CMC; purple, P80, light blue, water*—A. muciniphila*; light orange, CMC*—A. muciniphila*; light purple, P80*—A. muciniphila)*. (C, D) Unweighted Unifrac distance separating mice from different groups at (C) day 0 and (D) day 49. (E) Shannon alpha-diversity index at day 49. Data are represented as means±SEM. n=10–25 for the Unweighted Unifrac metric, and n=4–5 for the Shannon index. Statistical analyses were performed using a one-way ANOVA followed by a Bonferroni post hoc test and significant differences were recorded as follows: *p<0.05, **p<0.01, ***p<0.001. ANOVA, analysis of variance; CMC, carboxymethylcellulose; P80, polysorbate 80.

The impacts of emulsifiers on intestinal microbiota play a central role in promoting intestinal inflammation and its downstream consequences.^5 11^ Hence, we next examined the extent to which *A. muciniphila* might ameliorate emulsifier-induced changes in gut microbiota composition. Use of 16S sequencing followed by principal coordinate analysis (PCoA) of the unweighted Unifrac distances revealed that mice used here had homogeneous baseline microbiotas prior to the start of treatment (day 0, figure 3A). In contrast, such analysis showed that 7 weeks exposure to CMC or P80 resulted in clear treatment-based microbiota clustering (day 49, figure 3A, B) indicating that both CMC and P80 markedly shifted microbiota composition. This clear alteration in microbiota composition was confirmed by quantification of the unweighted Unifrac distance, exhibiting highly significant impact of CMC and P80 consumption on microbiota composition compared with water-treated mice (figure 3C, D). *A. muciniphila* administration, by itself, also clearly impacted microbiota composition with a clear distinct clustering (figure 3B) and a significant increase in unweighted Unifrac distance separating mice from these two groups (figure 3D), while no effect was observed on the microbiota alpha diversity (figure 3E). However, consumption of CMC and P80 amidst *A. muciniphila* administration had only slight impacts on microbiota composition. Specifically, all *A. muciniphila*-treated groups were observed to tightly cluster together, irrespective of emulsifier treatment while measure of unweighted Unifrac distance showed slight shifts that were much less than that induced by CMC and P80 in the absence of *A. muciniphila*, thus demonstrating that *A. muciniphila* almost fully prevented microbiota disturbances that were otherwise induced by consuming these emulsifiers. Finally, to ensure *A. muciniphila*-based clustering was not solely due to its DNA being excreted in faeces, unweighted Unifrac was computed after removing all Qiime2-generated amplicon sequence variants (ASVs) related to the Verrucomicrobia phylum (online supplemental figure S2). This approach had no impact on the above-described clustering, indicating that daily *A. muciniphila* had impacted the intestinal microbiota composition independently of its own phylum.

We next performed MaAsLin2 (Microbiome Multivariable Associations with Linear Models) analysis to identify features which are the more significantly impacted by emulsifier consumption (online supplemental figure S3), by comparing water-treated animals to CMC-treated or P80-treated animals.^21^ Among those, 2 belonged to the *Allobaculum* genus (online supplemental figure S3A, B), 2 to the *Clostridiaceae* family (online supplemental figure S3C, D), 10 to the S24-7 family (online supplemental figure S3E–N), 2 to the *Rikenellaceae* family (online supplemental figure S3O–P) and the rest to the *Turicibacter*, *Prevotella*, *Odoribacter* genera and *Ruminococcaceae* family (online supplemental figure S3Q–T). Interestingly, *A. muciniphila* administration prevented emulsifier consumption-induced alteration of most of these taxa, while only few differences were not restored to baseline (water-treatment) levels in the context of daily *A. muciniphila.* For example, various members of belonging to *S24-7* family were significantly increased by CMC and P80 consumption. In contrast, these OTUs were not altered by CMC or P80 amidst daily administration of *A. muciniphila* (online supplemental figure S3E–I). Moreover, we observed disappearance of a *Prevotella*-related feature in emulsifier-treated mice, while *A. muciniphila* administration fully prevented such depletion (online supplemental figure S3R). Hence, these data demonstrate that *A. muciniphila* is having a marked impact on intestinal microbiota composition that makes microbiota refractory to emulsifier-induced alterations.

### *A. muciniphila* administration prevents emulsifier-induced intestinal abnormalities and microbiota encroachment

**Figure 4:**
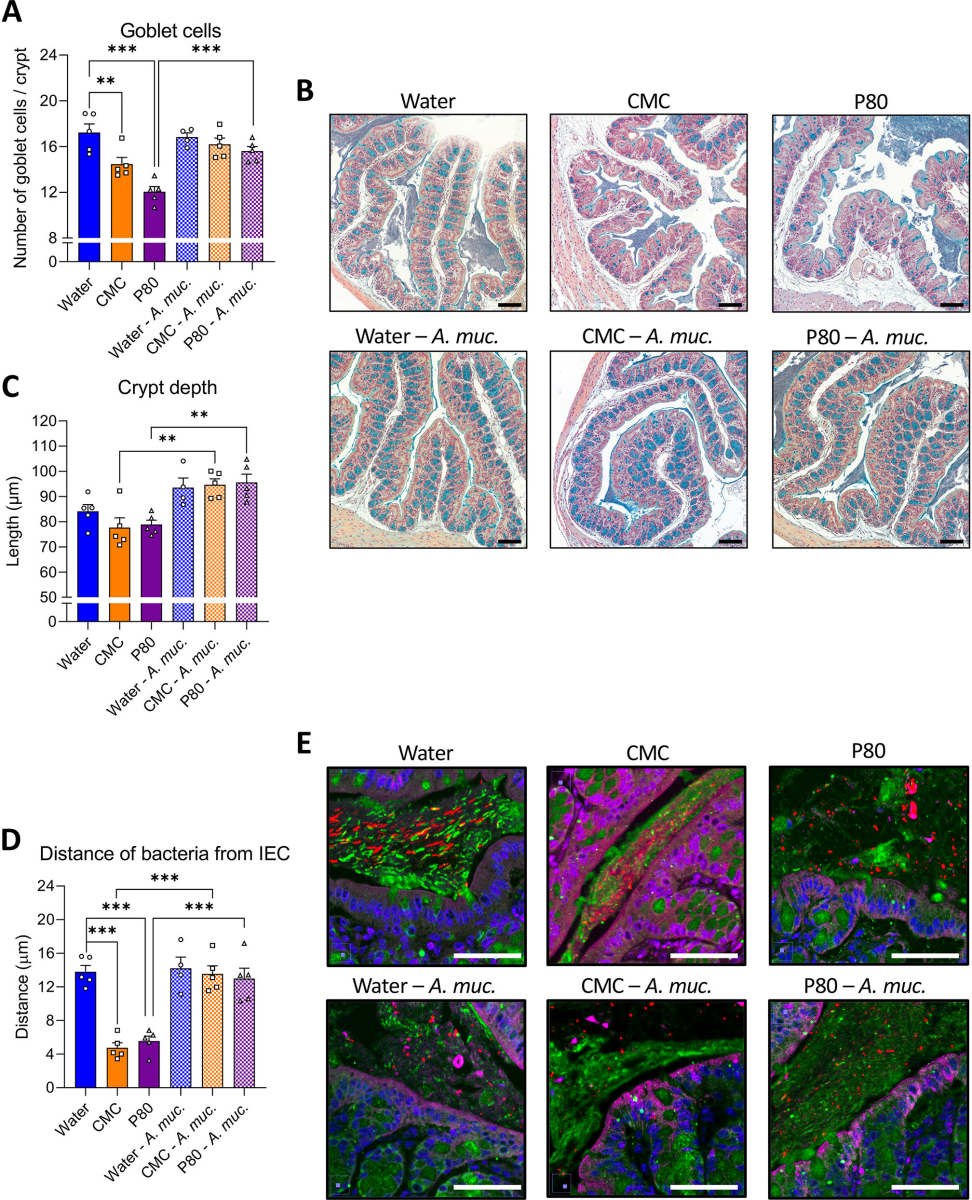
*Akkermansia muciniphila* administration prevents emulsifier-induced intestinal abnormalities and microbiota encroachment. Mice were exposed to drinking water (blue) containing 1.0% of CMC (orange) or P80 (purple) for 9 weeks, and gavaged 5 days per week with either vehicle (sterile PBS, solid bars) or *A. muciniphila* (*A. muc.*, hatched bars). Colon was subjected to immunostaining paired with fluorescent in situ hybridisation (FISH) followed by confocal microscopy analysis of microbiota localisation. (A, B) Colonic sections were stained with Alcian blue, and 17–23 crypts (3–5 per colonic sections) were randomly selected per animal to determine goblet cell number per crypt (A) as well as crypt depth (B). (C) Representative pictures obtained from 5 biological replicates, Alcian blue staining. Scale bar, 100 µm. (D) Distances of closest bacteria to intestinal epithelial cells (IEC) per condition over five high-powered fields per mouse. (E) Representative pictures obtained from 5 biological replicates, microbiota and mucus staining. n=4–5. MUC2, green; actin, purple; bacteria, red; and DNA, blue. Scale bar, 50 µm. Statistical analyses were performed using a one-way ANOVA followed by a Bonferroni post-hoc test and significant differences were recorded as follows: **p<0.01, ***p<0.001. ANOVA, analysis of variance; CMC, carboxymethylcellulose; P80, polysorbate 80.

Some changes in microbiota composition, including those induced by CMC and P80, can impact levels of proinflammatory agonists such as flagellin and LPS.^5^ Thus, we next measured functional levels of these agonists in faeces via use of TLR5 and TLR4 reporter cells. While a trend of emulsifiers resulting in elevated flagellin (FliC) and LPS was observed, it did not reach statistical significance (online supplemental figure S4A, B). Guided by previous studies,^22–24^ we sought colonic morphological alterations in animals consuming CMC and P80, and observed a decreased number of goblets cells per crypt (figure 4A, B). In contrast, animals receiving daily *A. muciniphila* administration were fully protected against emulsifier’s impact on goblet cells. Moreover, while emulsifier consumption alone was not sufficient to impact colonic crypt anatomy, *A. muciniphila*-treated mice exhibited increased crypt depth (figure 4A–C), as previously described.^22–24^ Another consequence of emulsifier consumption is to induce microbiota to penetrate the mucus later manifesting as a decrease in the epithelium/microbiota distance.^5 11 25^ Such microbiota encroachment is hypothesised to play a central role in emulsifier-induced chronic low-grade intestinal inflammation and metabolic dysregulations through the activation of various innate and adaptive immune signalling. Hence, we next examined microbiota encroachment by measuring the distance separating microbiota members from the surface of the epithelium using confocal imaging of Carnoy-fixed colon specimen. This approach recapitulated reports that both CMC or P80 consumption induce microbiota encroachment, with the average bacteria/epithelium being reduced from 13.80 µm in water-treated mice to 4.75 µm in CMC-treated mice (p<0.001) and 5.55 µm in P80-treated mice (<0.001) (figure 4D, E). Such microbiota encroachment was not associated with any impact on circulating immunoreactivity towards FliC and LPS (online supplemental figure S4C, D). *A. muciniphila* administration by itself did not alter bacterial-epithelial distance but, remarkably, *A. muciniphila* administration fully prevented emulsifier-induced microbiota encroachment, with distances of 14.21 µm, 13.56 µm and 12.99 µm being observed in water-treated, CMC-treated and P80-treated groups, respectively (figure 4D, E). Thus, *A. muciniphila* prevents emulsifier-induced microbiota encroachment, which is a cardinal feature of gut inflammation.

### *A. muciniphila* administration prevented emulsifier-induced alteration of the colonic transcriptome

**Figure 5:**
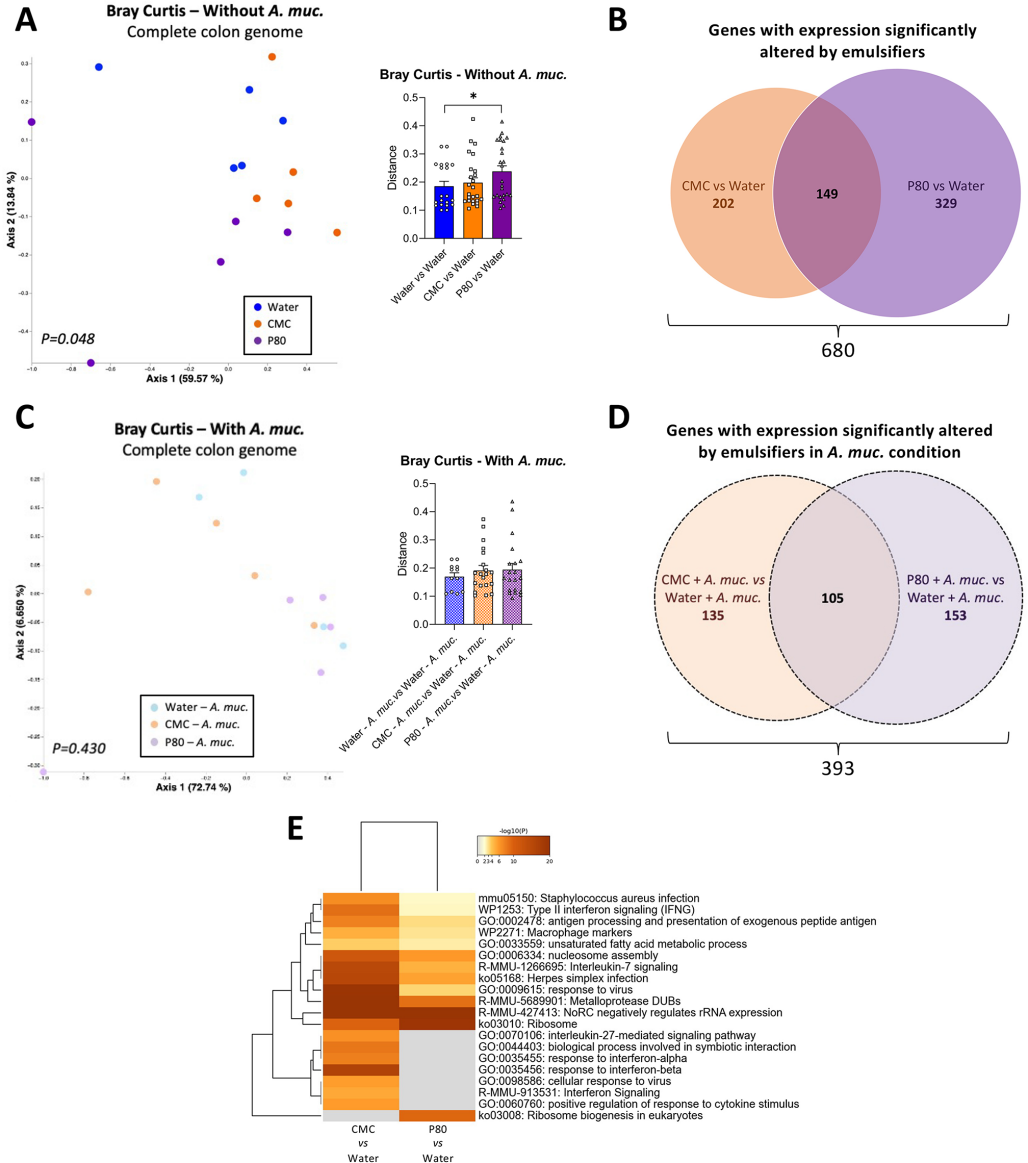
*Akkermansia muciniphila* administration prevented emulsifier-induced alteration of the colonic transcriptome. Mice were exposed to drinking water containing 1.0% of CMC or P80 for 9 weeks, and gavaged 5 days per week with either sterile PBS or *A. muciniphila* (*A. muc.*). Colon RNA was extracted and subjected to NextSeq sequencing. (A) Principal coordinates analysis (PCoA) of the Bray-Curtis distance matrix of the colonic transcriptome (all genes included) with dot coloured by treatment (water=blue; CMC=orange; P80=purple). Bray-Curtis distance separating samples from various group is also presented. (B) Venn diagram presenting the number of genes with significantly altered expression induced by CMC (orange) and/or P80 (purple). (C) PCoA of the Bray-Curtis distance matrix of the colonic transcriptome (all genes included) with dot coloured by treatment (water*—A. muciniphila*=light blue; CMC*—A. muciniphila*=light orange; P80—*A. muciniphila*=light purple). Bray-Curtis distance separating samples from various group is also presented. (D) Venn diagram presenting the number of genes with significantly altered expression induced by CMC (orange) and/or P80 (purple) in *A. muciniphila*-treated groups. (E) Heatmaps representing altered pathways/functions for CMC versus water and P80 versus water comparisons. PERMANOVA p values are indicated in the bottom of each PCoA. CMC, carboxymethylcellulose; P80, polysorbate 80.

We next examined the extent to which *A. muciniphila* amelioration of emulsifier-induced changes in microbiota composition would impact on intestinal gene expression. We performed untargeted RNA-seq analysis to identify the impact of emulsifier consumption on colonic gene expression, as well as the potential modulatory role played by *A. muciniphila* supplementation. As revealed by PCoA of the Bray Curtis distance using the entire RNA-seq dataset, we observed that both CMC and P80 consumption significantly impacted the colonic transcriptome (figure 5A, Permanova p=0.048), with CMC and P80 significantly altering expression of 351 and 478 genes, respectively (figure 5B, C, Cuffdiff cut-off q-value<0.05). We also observed an effect of *A. muciniphila* administration on colonic gene expression of water-only treated mice, with 296 significantly altered genes (online supplemental figure S5A), concurring with previous observations.^26 27^ Moreover, *A. muciniphila* supplementation drastically reduced, but not completely abrogate, emulsifier-induced transcriptome alteration (figure 5D and online supplemental figure S5) (Permanova p=0.430) (figure 5E). Deeper analysis of differentially expressed genes (DEGs) revealed that CMC and P80 induced shared and compound-specific alterations, with 202 genes impacted by CMC, 329 genes impacted by P80, and 149 genes impacted by both emulsifiers (figure 5B, online supplemental figure 5B, C, online supplemental tables S 2 and S 3). Interestingly, based on the number of variables studied and the percentage of impacted genes, only 8 common genes, instead of 149, should have been observed if CMC and P80 were impacting colon gene expression through independent mechanism, supporting that these two compounds drive common alterations—likely related to mucosal inflammation. This was further supported by PCoA of the Bray Curtis distances focusing on these genes, which displayed strong differential clustering between water-treated and emulsifier-treated groups along PC1, and to a less extent between CMC and P80 groups along PC2 (online supplemental figure S5F, Permanova p=0.006). Moreover, *A. muciniphila* supplementation fully prevented this clustering, as presented online supplemental figure S5G (Permanova p=0.150), indicating that *A. muciniphila* administration was able to counteract both CMC-induced and P80-induced transcriptomic alterations.

10.1136/gutjnl-2021-326835.supp2 Supplementary data

10.1136/gutjnl-2021-326835.supp3 Supplementary data

At the functional level, the DEGs altered in response to emulsifiers comprise an array of functions including inflammatory (macrophage markers, antigen processing and presentation, interleukin-7 and interleukin-27 signalling pathways, regulation of response to cytokine stimulus) and metabolic (unsaturated fatty acid metabolic process, regulation of lipid metabolic process secretion) processes (figure 5C and online supplemental figure S6). That daily *A. muciniphila* administration restored basal levels of expression for these genes and processes, as presented online supplemental figure S6, S7, further supported the notion that this bacterium promotes a healthy mucosal environment in contexts that normally associate with chronic intestinal inflammation and metabolic dysregulation.

## Discussion

Microbiota dysbiosis is thought to play a central role in driving intestinal inflammation and, consequently, numerous chronic inflammatory diseases.^28 29^ Features of microbiota dysbiosis include alterations in species composition with depletion of beneficial bacteria as well as microbiota encroachment, which is defined by increased bacterial penetrance into the normally near-sterile inner mucus layer.^3 5^ Such encroaching microbiota are thought to play an outsize role in driving gut inflammation.^3 5^ While there are likely a broad array of underlying causes for microbiota dysbiosis and encroachment, the increased incidence of chronic inflammatory diseases supports a major role for environmental (ie, non-genetic) determinants.^30^ For example, we and others have shown that consumption of dietary emulsifiers can induce altered microbiota composition and encroachment that most commonly results in low-grade inflammation and metabolic syndrome.^5 12 15 31 32^ Here, we examined a possible means of preventing such emulsifier-induced phenotypes, namely via direct administration of the mucus-fortifying bacteria *A. muciniphila,* which is depleted in metabolic syndrome and other chronic inflammatory diseases.^33–36^ We observed that endogenous *A. muciniphila* was depleted by emulsifiers, while administration of exogenous *A. muciniphila* fully prevented emulsifier impacts on microbiota and host. Specifically, the stark impacts of both CMC and P80 on microbiota composition, microbiota localisation, colon gene expression, inflammatory indices and metabolism were all absent in *A. muciniphila*-treated mice. Thus, *A. muciniphila* administration may be one means to avoid detrimental consequences of emulsifier consumption.

First isolated in 2004 by Derrien *et al*,^13^ this bacterium, present in mice and humans, has subsequently gained attention with the observation of its ability to prevent metabolic dysregulations in both preclinical and clinical studies.^16–18 20^ Mechanisms by which *A. muciniphila* benefits these disorders have not been entirely elucidated yet, but are thought to involve the ability of this mucus-loving bacterium to stimulate mucus production, potentially through its ability to digest it, but also most likely, by upregulating host defenses and mucus secretion *via* surface and/or secreted molecules, thus speeding mucus turnover, and potentially fortifying it.^17 37^ The mechanism by which *A. muciniphila* protects the intestine from emulsifiers remains to be defined but might involve membrane-associated Amuc_100,^17 38^ secreted P9,^19^ membrane-associated phospholipid diacyl phosphatidylethanolamine,^39^ and/or the ADP-heptose-like molecule, recently identified as being released by *A. muciniphila* with the ability to modulate the NF-kB signalling pathway.^40^ Moreover, accumulating evidence suggest that *A. muciniphila* interaction with the host involves TLR2-signalling pathways^17 39^ as well as modulation of IL10 and IL22 cytokines.^41 42^ Thus, future studies to identify the mechanism at play during protection against emulsifier-induced metabolic deregulations are warranted.^43^

The current study, together with previous reports that emulsifier can directly impact in vitro human microbiota,^11 12^ led us to hypothesise that *A. muciniphila* might prevent emulsifier-induced microbiota encroachment and its impacts on inflammation and metabolism without a direct impact on microbiota composition. However, *A. muciniphila* also surprisingly appears to prevent emulsifier-induced changes of microbiota composition. Hence, a possible explanation for our results is that the primary mechanism of action for *A. muciniphila* is indeed through the fortification of the mucus barrier, as suggested by its ability to reverse emulsifier-induced depletion in colonic goblet cells, and that, in vivo, altered microbiota composition is a consequence of encroachment-induced inflammation rather than the reflection of a direct emulsifier-microbiota interaction, which had been suggested by our in vitro studies. However, arguing against this possibility, we did not observe alteration in mucus gene expression in response to *A. muciniphila*, by itself or in presence of emulsifiers. Therefore, we propose an alternate and/or additional potential mechanism of *A. muciniphila* action. Specifically, we postulate that *A. muciniphila* might act directly on microbiota, shifting its composition to one that is resistant to emulsifier’s perturbation. Indeed, our data accords with this suggested mechanism, but further studies are needed to understand how *A. muciniphila* can possibly protect microbiota against emulsifiers. We envision use of ex vivo colonic explants to study the dynamic of mucus secretion and function,^44^ which, together with, longitudinal investigation of microbiota composition evolution during *A. muciniphila* supplementation, will elucidate impact of this probiotic on the mucus–microbiota relationship. Furthermore, it remains important to investigate the specificity of *A. muciniphila*-mediated protection by analysing the impacts of other commensal bacteria. This includes other microbiota members that are detrimentally impacted by emulsifier exposure, such as *Bifidobacterium*, *Prevotella* and *Faecalibacterium*,^5 10^ as well as bacterium with the ability to modulate mucus layer homoeostasis, such as *Bacteroides thetaiotaomicron*.^45^ Collectively, we anticipate these studies will yield mechanistic understanding of how *A. muciniphila* protects against dietary emulsifier consumption.

While this study primarily focused on *A. muciniphila*, in the course of studying its action, we also performed the first non-targeted study of emulsifier-induced microbiota encroachment impacts on colon transcriptome via RNA-seq analysis. This approach revealed profound host response induced by both emulsifiers, and while 22% of the deregulated genes were common between CMC-treated and P80-treated mice, 78% were specific to only one compound, supporting our previous observations that these two emulsifiers act through both common and specific mechanisms on the host–microbiota interface. Further in-dept characterisation of emulsifier-induced intestinal inflammation await investigation. For example, use of flow cytometry for immune cell phenotyping and/or single cell RNA-sequencing approaches to investigate the impact of emulsifier consumption on the transcriptome at the cell level should lead to a better understanding of the host response to emulsifier consumption, as well as the impact of *A. muciniphila* administration in this context.

While emulsifier-induced metabolic dysregulation serves as a tractable model to potential means of remediating dysbiosis, the protection afforded by *A. muciniphila* in this model may prove broadly applicable to other triggers of inflammation. Indeed, while CMC and P80 promote metabolic dysregulations in WT mice, they increase incidence and severity of colitis and cancer in mice genetically predisposed to these disorders.^5 15 25^ Regardless of what mechanism is ultimately ascribed to *A. muciniphila*’s protection against emulsifier-induced metabolic dysregulations, we predict it would likely extend to these other disorders. Moreover, we report here that similar protection was conferred by *A. muciniphila* on CMC or P80 exposure, both of which are non-metabolisable^46 47^ and act on the intestinal microbiota *via* different mechanisms,^11^ suggesting that the protection observed is not compound-specific. It nonetheless remains necessary to investigate the ability of *A. muciniphila* supplementation to protect against other additives, such as carrageenan emulsifier found to have a stark detrimental impact on microbiota composition and proinflammatory potential.^12^ Similarly, we would anticipate that either a more stable microbiota and/or a more rapidly renewing mucus layer might offer protection against a variety of modern stressors that might otherwise induce microbiota dysbiosis and, consequently, inflammation. This view accords with findings by Cani's laboratory and collaborators, founding that live or pasteurised *A. muciniphila* ameliorated metabolic parameters not only in mice but also in a small proof-of concept clinical trial in which the underlying metabolic syndrome of the study subjects can be presumed to have resulted from a variety of multifactorial underlying causes.^16–18 20^ That said, given the increasing recognised associations of consumption of ultraprocessed foods, which often contain multiple emulsifiers, we posit that consumers of such foods would be particularly likely to benefit from *A. muciniphila* supplementation. While designing a practical strategy to deliver such protection will require better understanding of underlying mechanism, it may ultimately prove to be a means of mitigating some of the negative aspects of these foods.

## Material and methods

### Materials

Sodium CMC (average MW ~250 000) and P80 were purchased from Sigma (Sigma, St. Louis, MO). *A. muciniphila* previously isolated by Derrien *et al* 13 was purchased from ATCC (Reference #BAA-835). Following ATCC recommendation, *A. muciniphila* was grown in Brain Heart Infusion broth for 72 hours at 37°C under strict anaerobic conditions. Bacteria were then pelleted by centrifugation 15 min at 4500 g, washed twice in sterile PBS, and aliquoted at 6.32×10^8^ bacteria per mL (determined by serial dilution and platting on Brain Heart Infusion agar plates) before storage at −80°C. The purity of the obtained aliquot was determined by bacterial DNA extraction and 16S rRNA gene sequencing, revealing the absence of environmental contamination in the *A. muciniphila* suspension (online supplemental figure S1A).

### Mice

The 5–6 week-old wild-type C57BL/6 male mice were purchased from The Jackson Laboratory (Reference # 000664). Mice were randomly grouped housed (n=5 per cage) at Georgia State University under institutionally approved protocol (Institutional Animal Care and Use Committee No A18006) and kept on LabDiet rodent chow diet #5001 ad libitum and reverse-osmosis treated Atlanta city water ad libitum. Mice were exposed to water (control group, N=10), CMC (N=10) or P80 (N=10) diluted in the drinking water (1.0% w/v and v/v, respectively) for 9 weeks, with solutions changed every week. For each group, half of the mice (n=5) were treated 5 days per week with 400 µL of sterile PBS (vehicle) and half of the mice (N=5) were treated 5 days per week with 400 µL of PBS containing 2.528×10^8^ colony-forming units of *A. muciniphila*. Body weights were measured every week. Food intake was measured twice during the same week by placing groups of mice in a clean cage with a known amount of food, for 24 hours, at which point the remaining food was weighted. Food consumption was expressed as gram per mouse per 24 hours. Fresh faeces were collected at days 0, 49 and 63 for downstream analysis. After 9 weeks of treatment (day 63), mice were euthanised, and one side of the epididymal fat pad weight, spleen weight, colon weight and colon length were measured. Tissues were collected for downstream analysis, as detailed below. The detailed experimental design is represented in figure 1B.

### Fasting blood glucose measurement

After 5 weeks of treatment, mice were placed in a clean cage and fasted for 15 hours. Blood glucose concentration was then determined using a Nova Max Plus Glucose Metre and expressed in mg/dL.

### Oral GTT

After 8 weeks of treatment, mice were 15 hours fasted and underwent a GTT. A bolus of glucose (2 g/kg of body weight) was intraperitoneally administered to the animals and glycaemia was recorded before the glucose challenge and after 15, 30, 60, 90, 120 min using a Nova Max plus Glucose metre.

### Quantification of faecal lipocalin-2 (Lcn-2) by ELISA

For quantification of faecal Lcn-2 by ELISA, frozen faecal samples were reconstituted in PBS containing 0.1% Tween 20 to a final concentration of 100 mg/mL and vortexed for 20 min to get a homogeneous faecal suspension.^48^ These samples were then centrifuged for 10 min at 14 000 g and 4°C. Clear supernatants were collected and stored at −20°C until analysis. Lcn-2 levels were estimated in the supernatants using Duoset murine Lcn-2 ELISA kit (R&D Systems, Minneapolis, Minnesota, USA) using the colorimetric peroxidase substrate tetramethylbenzidine, and optical density was read at 450 nm (Versamax microplate reader).

### Microbiota analysis by 16S rRNA gene sequencing using illumina technology

Microbiota analyses were performed before (day 0) and after (day 49) dietary emulsifier exposure. *A. muciniphila* relative abundance presented figure 1A were measured in a previous protocol, following 16 weeks of dietary emulsifier exposure. 16S rRNA gene amplification and sequencing were done using the Illumina MiSeq technology following the protocol of Earth Microbiome Project with their modifications to the MOBIO PowerSoil DNA Isolation Kit procedure for extracting DNA (https://press.igsb.anl.gov/earthmicrobiome).^49 50^ Bulk DNA was extracted from frozen faeces using a PowerSoil-htp kit from MoBio Laboratories (Carlsbad, California, USA) with mechanical disruption (bead-beating). The 16S rRNA genes, region V4, were PCR amplified from each sample using a composite forward primer and a reverse primer containing a unique 12-base barcode, designed using the Golay error-correcting scheme, which was used to tag PCR products from respective samples^50^). We used the forward primer 515F 5’- *AATGATACGGCGACCACCGAGATCTACACGCT*XXXXXXXXXXXXTATGGTAATT*GT* GTGYCAGCMGCCGCGGTAA-3’: the italicised sequence is the 5’ Illumina adaptor, the 12 X sequence is the golay barcode, the bold sequence is the primer pad, the italicised and bold sequence is the primer linker, and the underlined sequence is the conserved bacterial primer 515F. The reverse primer 806R used was 5’-*CAAGCAGAAGACGGCATACGAGAT*AGTCAGCCAG*CC* GGACTACNVGGGTWTCTAAT-3’: the italicised sequence is the 3’ reverse complement sequence of Illumina adaptor, the bold sequence is the primer pad, the italicised and bold sequence is the primer linker and the underlined sequence is the conserved bacterial primer 806R. PCR reactions consisted of Hot Master PCR mix (Quantabio, Beverly, Massachusetts, USA), 0.2 mM of each primer, 10–100 ng template and reaction conditions were 3 min at 95°C, followed by 30 cycles of 45 s at 95°C, 60 s at 50°C and 90 s at 72°C on a Biorad thermocycler. PCRs products were purified with Ampure magnetic purification beads (Agencourt, Brea, California, USA), and visualised by gel electrophoresis. Products were then quantified (BIOTEK Fluorescence Spectrophotometer) using Quant-iT PicoGreen dsDNA assay. A master DNA pool was generated from the purified products in equimolar ratios. The pooled products were quantified using Quant-iT PicoGreen dsDNA assay and then sequenced using an Illumina MiSeq sequencer (paired-end reads, 2×250 bp) at Cornell University, Ithaca.

### 16S rRNA gene sequence analysis

16S rRNA sequences were analysed using QIIME2—version 2019.^51^ Sequences were demultiplexed and quality filtered using the Dada2 method^52^ with QIIME2 default parameters in order to detect and correct Illumina amplicon sequence data, and a table of Qiime 2 artefact was generated. A tree was next generated, using the align-to-tree- mafft-fasttree command, for phylogenetic diversity analyses, and alpha and beta diversity analyses were computed using the core-metrics-phylogenetic command. PCoA plots were used to assess the variation between the experimental group (beta diversity). For taxonomy analysis, features were assigned to operational taxonomic units (OTUs) with a 99% threshold of pairwise identity to the Greengenes reference database 13_8.^53^ Unprocessed sequencing data are deposited in the Genome Sequence Archive (GSA) in BIG Data Centre, Beijing Institute of Genomics, Chinese Academy of Sciences, under accession number XXXXX, publicly accessible at http://bigd.big.ac.cn/gsa.

### Quantitative PCR analysis

Bacterial DNA was extracted from serially diluted *A. muciniphila* stock using the QIAamp Fast DNA Stool Mini kit, following manufacturer instruction (Qiagen). Quantitative PCR was subsequently performed on a LigthCycler 480 instrument (Roche Molecular Systems) on DNA from serially diluted *A. muciniphila* stock, as well as on DNA extracted from faecal samples collected at day 28. The QuantiFast SYBR Green PCR kit (Qiagen) was used with the following *A. muciniphila*-specific primers: forward *A. muciniphila*, CAGCACGTGAAGGTGGGGAC, reverse *A. muciniphila*, CCTTGCGGTTGGCTTCAGAT, as previously reported.^16^ Results are expressed in *A. muciniphila* /mg faeces based on a standard curve obtained from the serially diluted *A. muciniphila* stock.

### Staining of colonic tissue and histopathologic analysis

Mouse proximal colons were placed in methanol-Carnoy’s fixative solution (60% methanol, 30% chloroform, 10% glacial acetic acid) for a minimum of 3 hours at room temperature and stored at 4°C. Tissues were then washed in methanol 2×30 min, absolute ethanol 2×15 min, ethanol/xylene (1:1) 15 min and xylene 2×15 min, followed by embedding in Paraffin with a vertical orientation. Tissues were then sectioned at 4 µm thickness. For histological score, slides were stained with H&E using standard protocols. Images were acquired using a Lamina Slide Scanner (Perkin Elmer) at the Hist’IM platform (INSERM U1016, Paris, France). Histological scoring (ranging from 0 to 36) was blindly determined on each colon as previously described.^48 54^ Briefly, each colon was assigned four scores based on the degree of epithelial damage and inflammatory infiltrate in the mucosa, submucosa and muscularis/serosa.^54^ Each of the four scores was multiplied by a coefficient 1 if the change was focal, 2 if it was patchy and 3 if it was diffuse^48^ and the 4 individual scores per colon were added.

Colonic sections (4 µm) were also stained with Alcian Blue, preferentially staining mucopolysaccharides, and 17–23 crypts (3–5 per colonic sections) were randomly selected per animal to determine goblet cell number per crypt as well as crypt depth.

### Statistical analysis

Data are expressed as means±SEM and statistical analyses were performed using GraphPad Prism software (V.8). Significance was determined using a one-way analysis of variance (ANOVA), followed by a Bonferroni post hoc test for bar graphs and noted as follows: *p<0.05, **p<0.01, ***p<0.001. For data collected at different timepoints in line chart form, a two-way repeated measures ANOVA or a mixed-effects model (if missing values) with a Bonferroni post hoc test was performed and significance was noted as follows: CMC vs water, *p<0.05, **p<0.01, ***p<0.001; P80 vs water, ^#^p<0.05, ^##^p<0.01, ^###^p<0.001. Results were considered significant at p<0.05. For statistical analysis of microbiota data, the 20 most significantly differentially abundant features were identified using Microbiome Multivariable Associations with Linear Models (MaAsLin 2).^21^ Threshold for Volcano plots was set at q<0.05.

Please see online supplemental material for Supplementary Methods.

10.1136/gutjnl-2021-326835.supp5 Supplementary data

## Data Availability

Data are available upon reasonable request. Unprocessed sequencing data are deposited in the European Nucleotide Archive under accession number PRJEB57855.
