# Supplementary material for: Akkermansia muciniphila counteracts the deleterious effects of dietary emulsifiers on microbiota and host metabolism
Source: Gut. 2023 Jan 16;72(5):906–17. doi: 10.1136/gutjnl-2021-326835 (PMC10086484; doi:10.1136/gutjnl-2021-326835)
Supplement: Supplementary data [file gutjnl-2021-326835supp004.pdf]

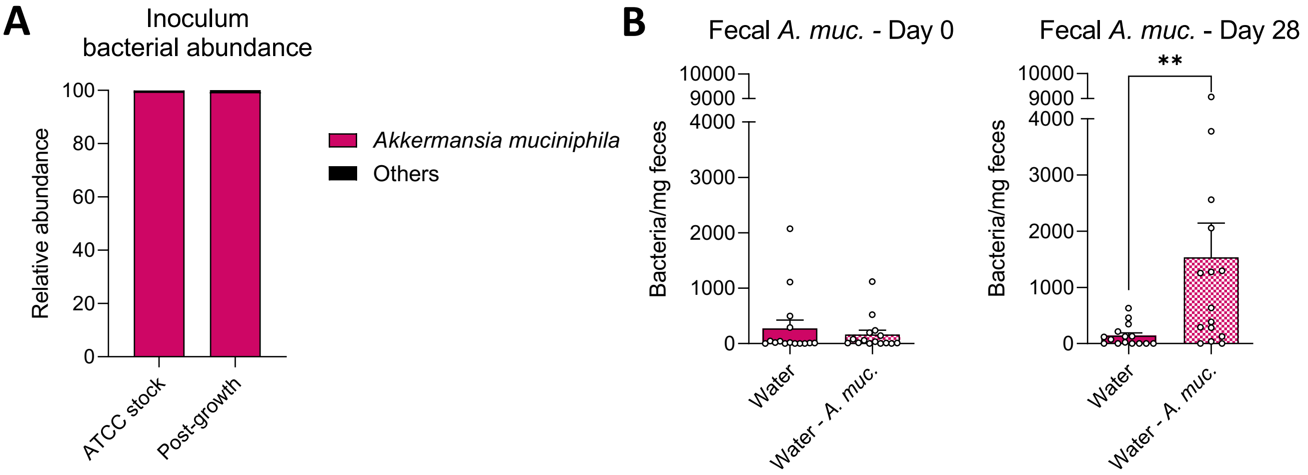

Supplementary Figure 1. Inoculum purity and impact of daily gavage on *A. muc.* fecal relative abundance. (**A**) Purity of the obtained *in vitro* bacterial stock was determined by bacterial DNA extraction, 16S rRNA gene sequencing, and Greengenes taxonomic assignment. (**B**) Fecal abundance was measured by qPCR in fecal DNA extraction from days 0 and 28 samples.

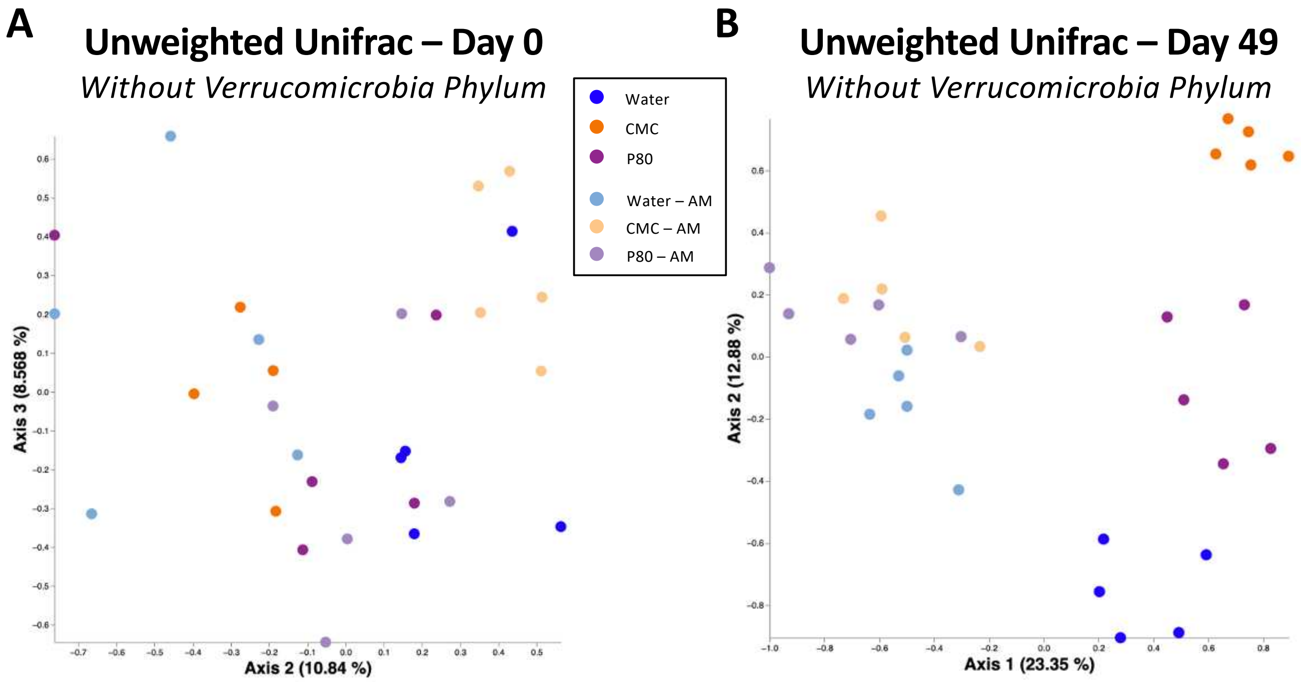

Supplementary Figure 2. *A. muc.* administration prevents emulsifier-induced alterations in microbiota composition. Principal coordinates analysis (PCoA) of the unweighted Unifrac matrix of microbiota assessed by 16S rRNA gene sequencing at days (**A**) 0 and (**B**) 49 after removing all Qiime2-generated ASVs related to the Verrucomicrobia phylum. Each dot represents an individual animal and is color coded (blue, water; orange, CMC; purple, P80, light blue, water – *A. muc.*; light orange, CMC – *A. muc.*; light purple, P80 – *A. muc.)*.

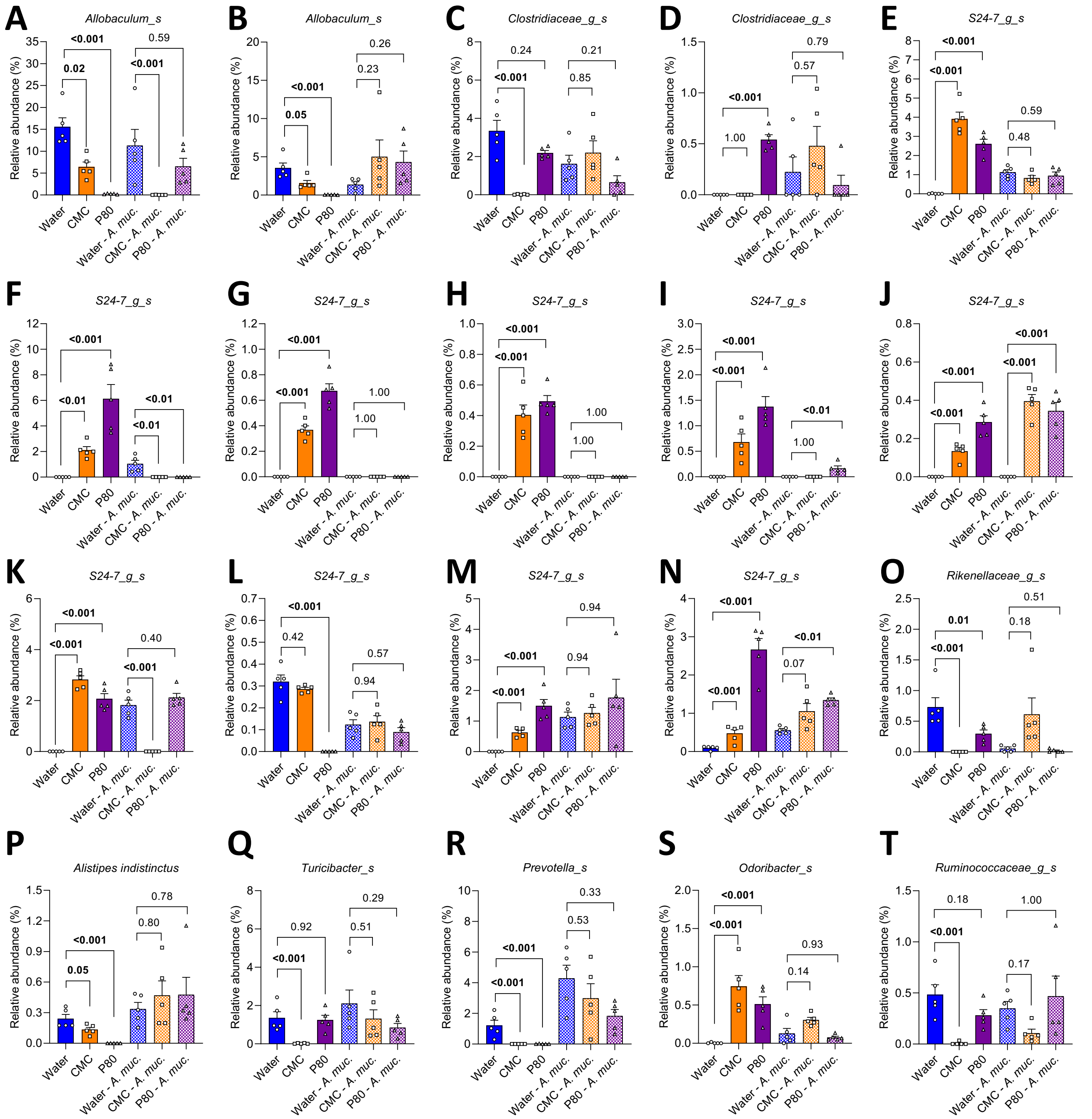

Supplementary Figure 3. *A. muc.* administration prevents dietary emulsifiers-induced microbiota alterations. Mice were exposed to drinking water (blue) containing 1.0% of CMC (orange) or P80 (purple) for 9 weeks, and gavaged 5 days per week with either sterile PBS (solid bars) or *A. muc.* (hatched bars). Fecal DNA was extracted at days 0 and 49 and subjected to 16S rRNA gene amplification and sequencing. The 20 most significantly differentially abundant features were identified using Microbiome Multivariable Associations with Linear Models (MaAsLin 2) and belonged to the (**A-B**) *Allobaculum* genus, (**C-D**) *Clostridiaceae* family, (**E-N**) *S24-7* family, (**O-P**) *Rikenellaceae* family, (**Q**) *Turicibacter* genus, (**R**) *Prevotella* genus, (**S**) *Odoribacter* genus and (**T**) *Ruminococcaceae* genus. Data are represented as means ± SEM. n=4-5. Statistical analyses were performed using MaAsLin 2. *P*-values of interest were directly recorded on graphs and significant differences are highlighted in bold.

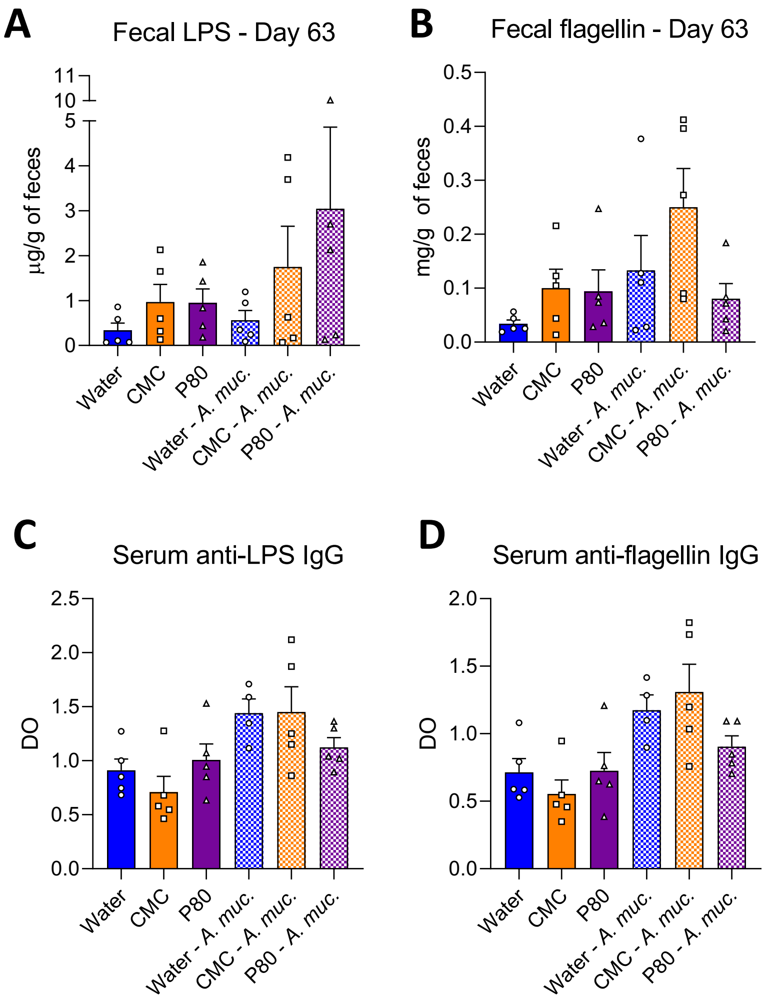

Supplementary Figure 4. Dietary emulsifiers and *A. muc.* administration impact on the intestinal environment. Mice were exposed to drinking water (blue) containing 1.0% of CMC (orange) or P80 (purple) for 9 weeks, and gavaged 5 days per week with either sterile PBS (solid bars) or *A. muc.* (hatched bars). Feces were collected at day 63 and (**A**) lipopolysaccharide (LPS) and (**B**) flagellin were measured using TLR4 and TLR5 reporter cells. Serum was collected at euthanasia and (**C**) anti-lipopolysaccharide (LPS) and (**D**) anti-flagellin IgG were measured. Data are represented as means ± SEM. n=4-5. Statistical analyses were performed using a one-way ANOVA followed by a Bonferroni post-hoc test and significant differences were recorded as follows: *p<0.05, **p<0.01.

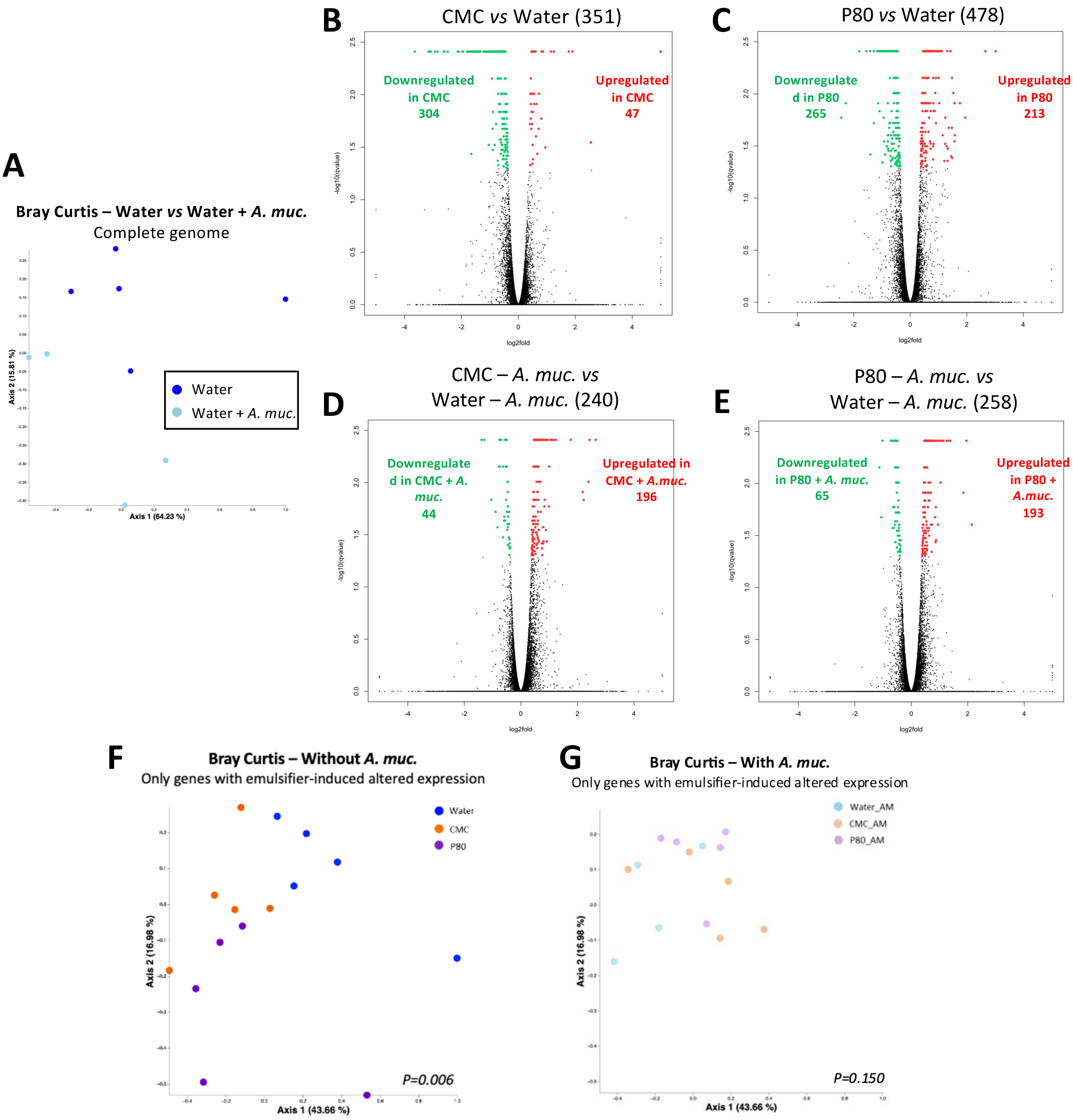

Supplementary Figure 5. *A. muc.* administration prevents emulsifier-induced alteration of the colonic transcriptome. Mice were exposed to drinking water containing 1.0% of CMC or P80 for 9 weeks, and gavaged 5 days per week with either sterile PBS or *A. muc.* Colon RNA was extracted and subjected to NextSeq sequencing. (**A**) Principal coordinates analysis (PCoA) of the Bray-Curtis distance matrix of the colonic transcriptome (all genes included) with dot colored by treatment (water = blue; water – *A. muc.* = light blue). (**B-C**) Colonic transcriptome at the gene level was visualized on volcano plots for CMC vs. water (**B**) and P80 vs. water (**C**) comparisons. For each gene, the difference in abundance between the two groups is indicated in Log2 fold change on the x-axis (with positive values corresponding to an increase in emulsifier-treated group compared with water-treated group, and negative values corresponding to a decrease in emulsifier-treated group compared with water-treated group), and significance between the two groups is indicated by −log10 q-value on the y-axis. (**D-E**) Colonic transcriptome at the gene level was visualized on volcano plots for CMC – *A. muc.* vs. water – *A. muc.* (**D**) and P80 – *A. muc.* vs. water – *A. muc.* (**E**) comparisons. (**F-G**) PCoA of the Bray-Curtis distance matrix for the genes with significantly altered expression induced by CMC and/or P80 with dot colored by treatment (water = blue; CMC = orange; P80 = purple; water – *A. muc.* = light blue; CMC – *A. muc.* = light orange; P80 – *A. muc.* = light purple). PERMANOVA p-values are indicated in the bottom of each PCoA.

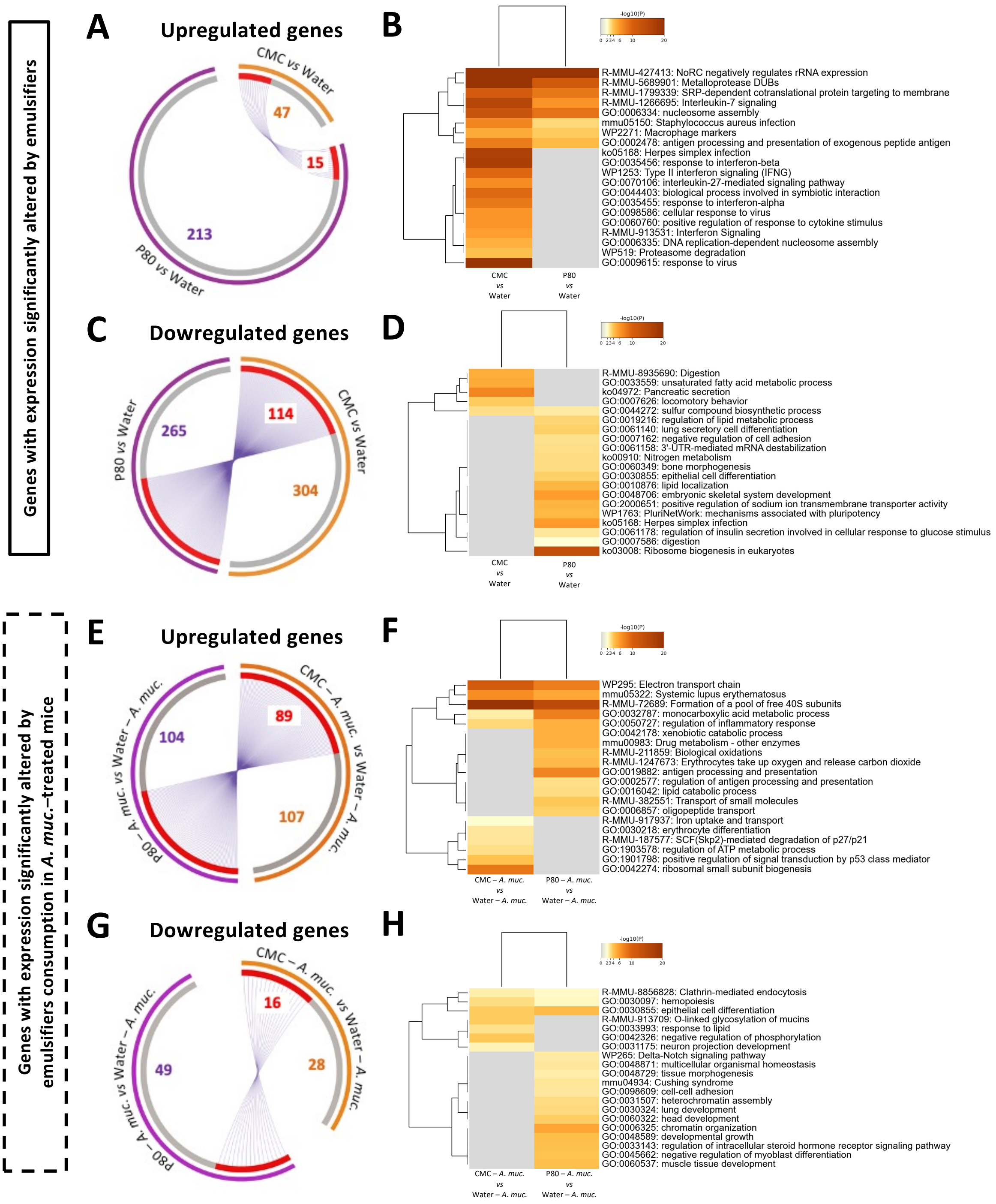

Supplementary Figure 6. Impact of emulsifier consumption and *A. muc.* treatment on the colonic transcriptome. (**A**) Overview of the number of genes with significantly increased expression following CMC or P80 consumption, with purple lines linking identical genes between CMC and P80 conditions. (**B**) Heatmaps listing overrepresented pathways / functions for CMC vs. Water and P80 vs. Water comparisons. (**C**) Overview of the number of genes with significantly decreased expression following CMC or P80 consumption, with purple lines linking identical genes between CMC and P80 conditions. (**D**) Heatmaps listing underrepresented pathways / functions for CMC vs. Water and P80 vs. Water comparisons. (**E**) Overview of the number of genes with significantly increased expression following CMC or P80 consumption in *A. muc.*-treated groups, with purple lines linking identical genes between CMC and P80 conditions. (**F**) Heatmaps listing overrepresented pathways / functions for CMC – *A. muc.* vs. Water – *A. muc.* and P80 – *A. muc.* vs. Water – *A. muc.* comparisons. (**G**) Overview of the number of genes with significantly decreased expression following CMC or P80 consumption in *A. muc.*-treated groups, with purple lines linking identical genes between CMC and P80 conditions. (**H**) Heatmaps listing underrepresented pathways / functions for CMC – *A. muc.* vs. Water – *A. muc.* and P80 – *A. muc.* vs. Water – *A. muc.* comparisons.

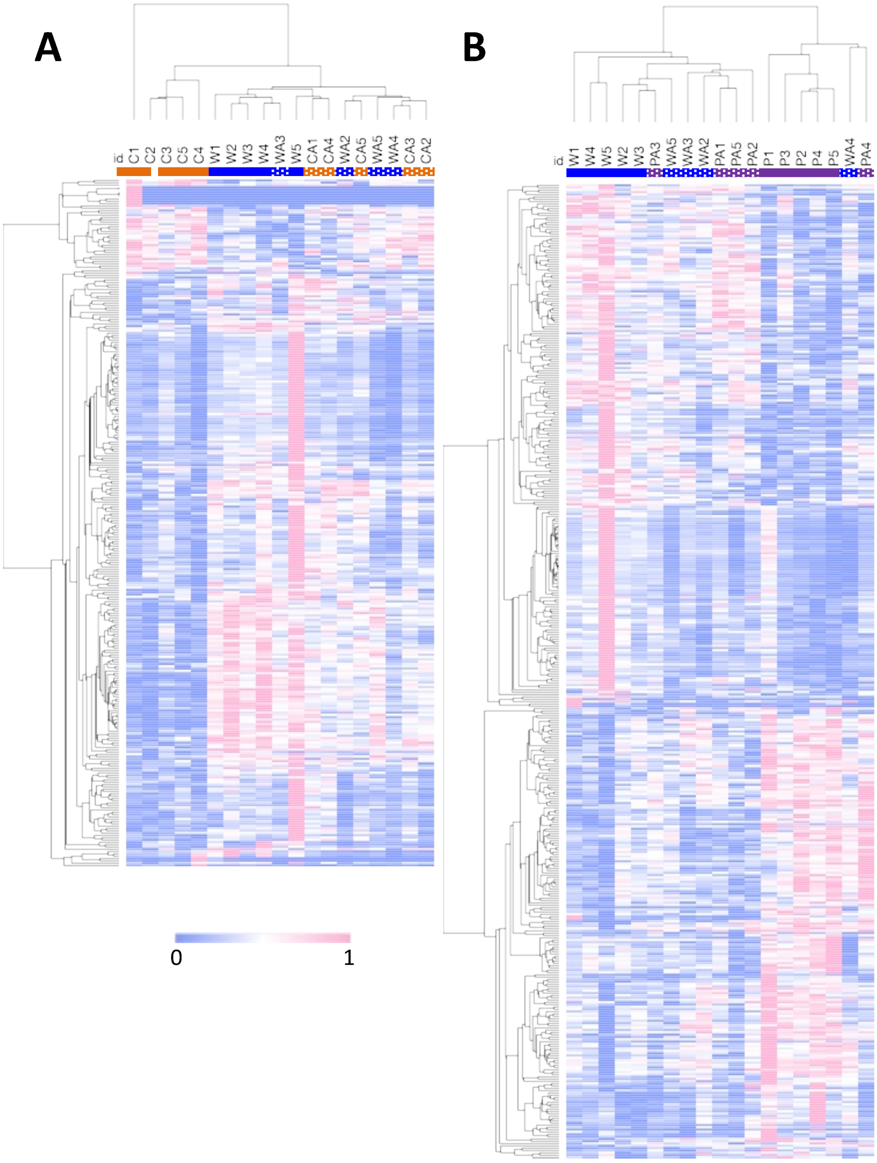

Supplementary Figure 7: Impact of emulsifier consumption and *A. muc.* treatment on colonic transcriptome. (**A**) Heatmap of genes with an altered expression induced by CMC consumption and for which *A. muc.* administration prevents such difference. (**B**) Heatmap of genes with an altered expression induced by P80 consumption and for which *A. muc.* administration prevents such difference. Hierarchical clustering was performed based on gene expression and Spearman rank correlations. W: water; C: CMC, WA: Water – *A. muc.*; CA: CMC – *A. muc.*; PA: P80 – *A. muc.*.
