# Supplementary material for: Akkermansia muciniphila counteracts the deleterious effects of dietary emulsifiers on microbiota and host metabolism
Source: Gut. 2023 Jan 16;72(5):906–17. doi: 10.1136/gutjnl-2021-326835 (PMC10086484; doi:10.1136/gutjnl-2021-326835)
Supplement: Supplementary data [file gutjnl-2021-326835supp005.pdf]

Supplementary Material

Supplementary Methods

Fecal flagellin and lipopolysaccharide load quantification

Levels of fecal bioactive flagellin and lipopolysaccharide (LPS) were quantified as previously described [1] using human embryonic kidney (HEK)-Blue-mTLR5 and HEK-BluemTLR4 cells, respectively (Invivogen, San Diego, CA, USA) [1]. Fecal material was resuspended in PBS to a final concentration of 100 mg/mL and homogenized for 10 s using a Mini-Beadbeater-24 without the addition of beads to avoid bacteria disruption. Samples were then centrifuged at 8000 g for 2 min and the resulting supernatant was serially diluted and applied on mammalian cells. Purified E. coli flagellin and LPS (Sigma-Aldrich) were used for standard curve determination using HEK-Blue-mTLR5 and HEK-Blue-mTLR4 cells, respectively. After 24 h of stimulation, the cell culture supernatant was applied to QUANTI-Blue medium (Invivogen) and the alkaline phosphatase activity was measured at 620 nm after 30 min.

Serum immunoreactivity to LPS and flagellin

Serum immunoreactivity to LPS and flagellin were examined by ELISA as described previously[2]. High-binding ELISA plates were coated overnight with purified flagellin (100 ng/well; SRP8029-10UG, Sigma) or LPS (2 µg/well; from E. coli 0128: B12, Sigma) in 9.6 pH bicarbonate buffer. Sera were diluted 1:100 and added to wells coated with flagellin or LPS. After incubation at 37°C for 1h, the wells were washed and then incubated with HRP-conjugated anti-mouse IgG (1:1000). After washing, the peroxidase substrate tetramethylbenzidine (TMB) was added to the wells and, after 5 min, optical density (OD) was read at 450 nm with an ELISA plate reader. Data are reported as OD corrected by subtracting with the readings in blank samples.

Colonic mRNA extraction

Distal colon was collected during euthanasia and placed in RNA-Later (Invitrogen). Total mRNAs were isolated from colonic tissues homogenized with TRIzol (Invitrogen, Carlsbad, CA) according to the manufacturer's instructions and as previously described[3]. Briefly, chloroform was then added, and samples were incubated at RT for 3min. After centrifugation (12,000g, 4°C, 15min), the aqueous phase was transferred in a new tube. RNA was precipitated by adding isopropyl alcohol and incubated overnight at -20°C. Samples were then centrifuged (12,000, 4°C, 30min), supernatant was aspirated, and pellet washed with 75% ethanol. Ethanol was then eliminated after centrifugation (7,500, 4°C, 5min) and dried RNAs were dissolved in molecular grade water. RNAs were subsequently cleaned using the RNeasy Mini Kit (Qiagen) according to the manufacturer's instructions. Extracted purified RNAs were quantified, and purity assessed using a Thermo Scientific™ Nanodrop™ one.

Colonic RNA sequencing

Library preparation and sequencing. cDNA library was prepared using the Invitrogen™ Collibri™ Stranded RNA library Prep Kit for Illumina™ with Collibri™ H/M/R rRNA Depletion Kit according to the manufacturer's instructions and starting with 500ng of purified RNAs. Briefly, rRNA were first depleted, and enriched mRNAs subsequently used for fragmentation, adaptors ligation and reverse transcription. After purification, libraries were PCR-enriched, further purified, and quantified and quality-assessed on an Agilent™ 2100 Bioanalyzer™ instrument. A master library was generated from the purified products in equimolar ratios. The pooled products were quantified using Qubit and sequenced using an Illumina Next-Seq sequencer (paired-end reads, 2x750 bp) at Cornell University, Ithaca.

Data analysis. Cutadapt tool [4] online tool was first used in order to remove adapter sequences s as well as trim sequences from the first low quality (<28) base. High quality reads longer than 20 nucleotides were then aligned to mm10 *mus musculus* reference genome using Bowtie2 [5]. Gene expression levels were next measured using Cufflinks [6] and differentially expressed genes between conditions were identified using Cuffdiff [6]. Fragments Per Kilobase of transcript per Million mapped reads (FPKM) unit was used and Log2 fold changes and q-values were computed for each comparison of interest. Principal coordinates analysis (PCoA) of the Bray-Curtis distance matrix of the colonic transcriptome were then generated for comparisons of interest. Gene level volcano plots were generated through R (version 4.1.2 (2021-11-01), Platform: x86_64-apple-darwin17.0 (64-bit)). Differentially expressed genes enrichment analysis was performed using Metascape (https://metascape.org/gp/index.html#/main/step1) [7]. User-provided gene identifiers were converted into their corresponding *Mus musculus* gene IDs using the last version of the database (last updated on 2021-11-01). Briefly, functions and metabolic pathways were ordered according to their *P*-value (smaller *P*-value indicating higher ranking). For each given gene list, pathway and process enrichment analysis were performed with the following ontology sources: GO Biological Processes, KEGG Pathway, Reactome Gene Sets, CORUM, TRRUST, PaGenBase and WikiPathways. Terms ($P<0.01$, minimum count of 3, enrichment factor>1.5) were collected and grouped into clusters based on their membership similarities. More specifically, *P*-values were calculated based on the accumulative hypergeometric distribution, and q-values were calculated using the Benjamini-Hochberg procedure to account for multiple testing. Kappa scores were used as the similarity metric when performing hierarchical clustering on the enriched terms, and sub-trees with a similarity of > 0.3 were considered a cluster. The most statistically significant term within a cluster was chosen to represent the cluster. Heatmaps were generated using Morpheus (https://software.broadinstitute.org/morpheus) online tool using 297 and 421 genes, respectively.

BMJ Publishing Group Limited (BMJ) disclaims all liability and responsibility arising from any reliance placed on this supplemental material which has been supplied by the author(s)

They were then clustered by rows and columns using the average linkage hierarchical clustering and Spearman rank correlations. These genes were selected based on the following criteria 1) significantly different between mice receiving dietary emulsifier and mice receiving water only and 2) not common between C vs. W and CA vs. WA, or P vs. W and PA vs. WA, in order to focus on differences vanishing in the *A. muc.*-treated groups. Unprocessed sequencing data are deposited in the Genome Sequence Archive (GSA) in BIG Data Center, Beijing Institute of Genomics, Chinese Academy of Sciences, under accession number XXXXX, publicly accessible at http://bigd.big.ac.cn/gsa.

Immunostaining of mucins and localization of bacteria by FISH

Mucus immunostaining was paired with fluorescent in situ hybridization (FISH), as previously described[8], in order to analyze bacteria localization at the surface of the intestinal mucosa[9,10]. Briefly, colonic tissues (proximal colon, 2nd cm from the cecum) containing fecal material were placed in methanol-Carnoy's fixative solution (60% methanol, 30% chloroform, 10% glacial acetic acid) for a minimum of 3 h at room temperature and stored at 4°C. Tissues were then washed in methanol 2x30 min, absolute ethanol 2x15 min, ethanol/xylene (1:1) 15 min and xylene 2x15 min, followed by embedding in Paraffin with a vertical orientation. Four mm sections were performed and dewax by xylene 60°C for 10 min, xylene for 10 min and 99.5% ethanol for 5 minutes. Hybridization step was performed at 50°C overnight with EUB338 probe (50-GCTGCCTCCCGTAGGAGT-30, with a 5' labeling using Alexa 647) diluted to a final concentration of 10 mg/mL in hybridization buffer (20 mM Tris–HCl, pH 7.4, 0.9 M NaCl, 0.1% SDS, 20% formamide). After washing 10 min in wash buffer (20 mM Tris–HCl, pH 7.4, 0.9 M NaCl) and a quick wash in PBS, slides were incubated in block solution (5% fetal bovine serum in PBS) in darkness at 4°C for 30 min. Slides were then gently dried and PAP pen (Sigma-Aldrich)

was used to mark around the section. Mucin-2 primary antibody (rabbit MUC2 antibody [C3], C-term, Genetex, GTX100664) was diluted 1:100 in block solution and applied overnight at 4°C. After washing 3x10 min in PBS, block solution containing anti-rabbit Alexa 488 secondary antibody diluted 1:300, Phalloidin-Tetramethylrhodamine B isothiocyanate (Sigma-Aldrich) at 1 mg/mL and Hoechst 33258 (Sigma-Aldrich) at 10 mg/mL was applied to the section for 2h. After washing 3x10 min in PBS slides were mounted using Prolong anti-fade mounting media (Life Technologies, Carlsbad, CA, USA) and kept in the dark at 4°C. Observations and measurement of the distance between bacteria and epithelial cell monolayer were performed with a Spinning Disk IXplore using the Olympus cellSens imaging software 421 (V2.3) at a frame size of 2,048 x 2,048 with 16-bit depth. A 405nm laser was used to excite the 422 Hoechst stain (epithelial DNA), 488nm for Alexa Fluor 488 (mucus), 488nm for TRITC (actin), 423 and 640nm for Alexa Fluor 647 (bacteria). Samples were imaged with a 20x objective.

Supplementary references

1 Chassaing B, Koren O, Carvalho FA, *et al.* AIEC pathobiont instigates chronic colitis in susceptible hosts by altering microbiota composition. *Gut* 2014;**63**:1069–80. doi:10.1136/gutjnl-2013-304909

2 Singh V, Yeoh BS, Chassaing B, *et al.* Dysregulated Microbial Fermentation of Soluble Fiber Induces Cholestatic Liver Cancer. *Cell* 2018;**175**:679-694.e22. doi:10.1016/j.cell.2018.09.004

3 Chassaing B, Srinivasan G, Delgado MA, *et al.* Fecal Lipocalin 2, a Sensitive and Broadly Dynamic Non-Invasive Biomarker for Intestinal Inflammation. *PLOS ONE* 2012;**7**:e44328. doi:10.1371/journal.pone.0044328

4 Martin M. Cutadapt removes adapter sequences from high-throughput sequencing reads. *EMBnet.journal* 2011;**17**:10–2. doi:10.14806/ej.17.1.200

5 Langmead B, Salzberg SL. Fast gapped-read alignment with Bowtie 2. *Nat Methods* 2012;**9**:357–9. doi:10.1038/nmeth.1923

6 Trapnell C, Roberts A, Goff L, *et al.* Differential gene and transcript expression analysis of RNA-seq experiments with TopHat and Cufflinks. *Nat Protoc* 2012;**7**:562–78. doi:10.1038/nprot.2012.016

7 Zhou Y, Zhou B, Pache L, *et al.* Metascape provides a biologist-oriented resource for the analysis of systems-level datasets. *Nat Commun* 2019;**10**:1523. doi:10.1038/s41467-019-09234-6

8 Johansson MEV, Hansson GC. Preservation of mucus in histological sections, immunostaining of mucins in fixed tissue, and localization of bacteria with FISH. *Methods Mol Biol Clifton NJ* 2012;**842**:229–35. doi:10.1007/978-1-61779-513-8_13

9 Chassaing B, Ley RE, Gewirtz AT. Intestinal Epithelial Cell Toll-like Receptor 5 Regulates the Intestinal Microbiota to Prevent Low-Grade Inflammation and Metabolic Syndrome in Mice. *Gastroenterology* 2014;**147**:1363-1377.e17. doi:10.1053/j.gastro.2014.08.033

10 Chassaing B, Koren O, Goodrich JK, *et al.* Dietary emulsifiers impact the mouse gut microbiota promoting colitis and metabolic syndrome. *Nature* 2015;**519**:92–6. doi:10.1038/nature14232

Supplementary figure legends

Supplementary Figure 1. Inoculum purity and impact of daily gavage on *A. muc.* fecal relative abundance. (**A**) Purity of the obtained *in vitro* bacterial stock was determined by bacterial DNA extraction, 16S rRNA gene sequencing, and Greengenes taxonomic assignment. (**B**) Fecal abundance was measured by qPCR in fecal DNA extraction from days 0 and 28 samples.

Supplementary Figure 2. *A. muc.* administration prevents emulsifier-induced alterations in microbiota composition. Principal coordinates analysis (PCoA) of the unweighted Unifrac matrix of microbiota assessed by 16S rRNA gene sequencing at days (**A**) 0 and (**B**) 49 after removing all Qiime2-generated ASVs related to the Verrucomicrobia phylum. Each dot represents an individual animal and is color coded (blue, water; orange, CMC; purple, P80, light blue, water – *A. muc.*; light orange, CMC – *A. muc.*; light purple, P80 – *A. muc.)*.

Supplementary Figure 3. *A. muc.* administration prevents dietary emulsifiers-induced microbiota alterations. Mice were exposed to drinking water (blue) containing 1.0% of CMC (orange) or P80 (purple) for 9 weeks, and gavaged 5 days per week with either sterile PBS (solid bars) or *A. muc.* (hatched bars). Fecal DNA was extracted at days 0 and 49 and subjected to 16S rRNA gene amplification and sequencing. The 20 most significantly differentially abundant features were identified using Microbiome Multivariable Associations with Linear Models (MaAsLin 2) and belonged to the (**A-B**) Allobaculum genus, (**C-D**) Clostridiaceae family, (**E-N**) S24-7 family, (**O-P**) Rikenellaceae family, (**Q**) Turicibacter genus, (**R**) Prevotella genus, (**S**) Odoribacter genus and (**T**) Ruminococcaceae genus. Data are represented as means ± SEM. n=4-

5. Statistical analyses were performed using MaAsLin 2. *P*-values of interest were directly recorded on graphs and significant differences are highlighted in bold.

Supplementary Figure 4. Dietary emulsifiers and *A. muc.* administration impact on the intestinal environment. Mice were exposed to drinking water (blue) containing 1.0% of CMC (orange) or P80 (purple) for 9 weeks, and gavaged 5 days per week with either sterile PBS (solid bars) or *A. muc.* (hatched bars). Feces were collected at day 63 and (**A**) lipopolysaccharide (LPS) and (**B**) flagellin (FliC) were measured using TLR4 and TLR5 reporter cells. Serum was collected at euthanasia and (**C**) anti-lipopolysaccharide (LPS) and (**D**) anti-flagellin (FliC) IgG were measured. Data are represented as means ± SEM. n=4-5. Statistical analyses were performed using a one-way ANOVA followed by a Bonferroni post-hoc test and significant differences were recorded as follows: *$p<0.05$, **$p<0.01$.

Supplementary Figure 5. *A. muc.* administration prevents emulsifier-induced alteration of the colonic transcriptome. Mice were exposed to drinking water containing 1.0% of CMC or P80 for 9 weeks, and gavaged 5 days a week with either sterile PBS or *A. muc.* Colon RNA was extracted and subjected to NextSeq sequencing. (**A-B**) Colonic transcriptome at the gene level was visualized on volcano plots for CMC vs. water (**A**) and P80 vs. water (**B**) comparisons. For each gene, the difference in abundance between the two groups is indicated in Log2 fold change on the x-axis (with positive values corresponding to an increase in emulsifier-treated group compared with water-treated group, and negative values corresponding to a decrease in emulsifier-treated group compared with water-treated group), and significance between the two groups is indicated by −log10 q-value on the y-axis. (**C-D**) Colonic transcriptome at the gene level was

visualized on volcano plots for CMC – *A. muc.* vs. water – *A. muc.* (**C**) and P80 – *A. muc.* vs. water – *A. muc.* (**D**) comparisons. (**E-F**) PCoA of the Bray-Curtis distance matrix for the genes with significantly altered expression induced by CMC and/or P80 with dot colored by treatment (water = blue; CMC = orange; P80 = purple; water – *A. muc.* = light blue; CMC – *A. muc.* = light orange; P80 – *A. muc*. = light purple). PERMANOVA p-values are indicated in the bottom of each PCoA.

Supplementary Figure 6. Impact of emulsifier consumption and *A. muc.* treatment on the colonic transcriptome. (**A**) Overview of the number of genes with significantly increased expression following CMC or P80 consumption, with purple lines linking identical genes between CMC and P80 conditions. (**B**) Heatmaps listing overrepresented pathways / functions for CMC vs. Water and P80 vs. Water comparisons. (**C**) Overview of the number of genes with significantly decreased expression following CMC or P80 consumption, with purple lines linking identifcal genes between CMC and P80 conditions. (**D**) Heatmaps listing underrepresented pathways / functions for CMC vs. Water and P80 vs. Water comparisons. (**E**) Overview of the number of genes with significantly increased expression following CMC or P80 consumption in *A. muc.*-treated groups, with purple lines linking identical genes between CMC and P80 conditions. (**F**) Heatmaps listing overrepresented pathways / functions for CMC + *A. muc.* vs. Water + *A. muc.* and P80 + *A. muc.* vs. Water + *A. muc.* comparisons. (**G**) Overview of the number of genes with significantly decreased expression following CMC or P80 consumption in *A. muc.*-treated groups, with purple lines linking identifcal genes between CMC and P80 conditions. (**H**) Heatmaps listing underrepresented pathways / functions for CMC + *A. muc.* vs. Water + *A. muc.* and P80 + *A. muc.* vs. Water + *A. muc.* comparisons.

Supplementary Figure 7: Impact of emulsifier consumption and *A. muc.* treatment on colonic transcriptome. (**A**) Principal coordinates analysis (PCoA) of the Bray-Curtis distance matrix of the colonic transcriptome (all genes included) with dot colored by treatment (water = blue; water + *A. muc.* = light blue) (**B**) Heatmap of genes with an altered expression induced by CMC consumption and for which *A. muc.* administration prevents such difference. (**C**) Heatmap of genes with an altered expression induced by P80 consumption and for which *A. muc.* administration prevents such difference. Hierarchical clustering was performed based on gene expression and Spearman rank correlations. W: water; C: CMC, WA: Water + *A. muc.*; CA: CMC + *A. muc.*; PA: P80 + *A. muc.*.
